# Secreted CLIC3 drives cancer progression through its glutathione-dependent oxidoreductase activity

**DOI:** 10.1038/ncomms14206

**Published:** 2017-02-15

**Authors:** Juan R. Hernandez-Fernaud, Elena Ruengeler, Andrea Casazza, Lisa J. Neilson, Ellie Pulleine, Alice Santi, Shehab Ismail, Sergio Lilla, Sandeep Dhayade, Iain R. MacPherson, Iain McNeish, Darren Ennis, Hala Ali, Fernanda G. Kugeratski, Heba Al Khamici, Maartje van den Biggelaar, Peter V.E. van den Berghe, Catherine Cloix, Laura McDonald, David Millan, Aoisha Hoyle, Anna Kuchnio, Peter Carmeliet, Stella M. Valenzuela, Karen Blyth, Huabing Yin, Massimiliano Mazzone, Jim C. Norman, Sara Zanivan

**Affiliations:** 1 Cancer Research UK Beatson Institute, Glasgow G611BD, UK; 2 Laboratory of Molecular Oncology and Angiogenesis, Vesalius Research Center, VIB, Leuven B-3000, Belgium; 3 Division of Biomedical Engineering, School of Engineering, University of Glasgow, Glasgow G12 8LT, UK; 4 Wolfson Wohl Cancer Research Centre, Institute of Cancer Sciences, University of Glasgow, Glasgow G611QH, UK; 5 School of Life Sciences, University of Technology Sydney, Sydney, New South Wales 2007, Australia; 6 Centre for Health Technologies, University of Technology Sydney, Sydney, New South Wales 2007, Australia; 7 Department of Plasma Proteins, Sanquin Research, Amsterdam 1066 CX, The Netherlands; 8 Department of Pathology, Queen Elizabeth University Hospital, Glasgow G51 4TF, UK; 9 Laboratory of Angiogenesis and Vascular Metabolism, VIB Center for Cancer Biology, Vesalius Research Center, VIB, B-3000 Leuven, Belgium

## Abstract

The secretome of cancer and stromal cells generates a microenvironment that contributes to tumour cell invasion and angiogenesis. Here we compare the secretome of human mammary normal and cancer-associated fibroblasts (CAFs). We discover that the chloride intracellular channel protein 3 (CLIC3) is an abundant component of the CAF secretome. Secreted CLIC3 promotes invasive behaviour of endothelial cells to drive angiogenesis and increases invasiveness of cancer cells both *in vivo* and in 3D cell culture models, and this requires active transglutaminase-2 (TGM2). CLIC3 acts as a glutathione-dependent oxidoreductase that reduces TGM2 and regulates TGM2 binding to its cofactors. Finally, CLIC3 is also secreted by cancer cells, is abundant in the stromal and tumour compartments of aggressive ovarian cancers and its levels correlate with poor clinical outcome. This work reveals a previously undescribed invasive mechanism whereby the secretion of a glutathione-dependent oxidoreductase drives angiogenesis and cancer progression by promoting TGM2-dependent invasion.

## Abstract (web summary)

The secretome from cancer and stromal cells contributes to the creation of a microenvironment, which in turn contributes to invasion and angiogenesis. Here, the authors compare the secretomes of immortalized normal fibroblasts and cancer-derived fibroblast and identify CLIC3 as a driver of cancer progression.

Acquisition of invasive characteristics by cancer cells is a watershed in the transition between indolent tumours (such as ductal carcinoma *in situ* (DCIS)), which are surrounded by an intact basement membrane, and more aggressive invasive carcinoma in which the basement membrane is disrupted. In addition, the invasive characteristics of vascular endothelial cells allow them to penetrate the tumour stroma to supply oxygen and nutrients that support cancer growth and provide a route for cancer cells to leave the tumour to form metastases^1^,^2^. The composition and physical properties of the microenvironment change dramatically during tumour development and the secretome of both stromal and cancer cells plays pivotal roles in this^3^,^4^. For example, the lysyl oxidase (LOX), which is released from cancer and stromal cells, promotes γ-lysyl cross-bridges to stiffen the extracellular matrix (ECM). This influences integrin signalling and promotes invasive behaviour of endothelial and cancer cells through β1 integrin-dependent signalling^5^,^6^. Inhibition of LOX decreases tumour angiogenesis and growth and opposes metastasis^6^,^7^,^8^, thus exemplifying the efficacy of strategies aimed at targeting secreted factors that alter the tumour microenvironment. In addition, the secretion of factors such as the transforming growth factor-β (TGFβ) and sonic hedgehog by cancer cells is now well established to lead to generation of populations of cancer-associated fibroblasts (CAFs) with an activated myofibroblast-like phenotype^9^,^10^. CAFs are abundant in the stroma of carcinomas and are a key contributing factor in the generation of an aberrant tumour microenvironment permissive for cancer progression^9^,^11^,^12^,^13^. Indeed, the secretion of soluble factors such as TGFβ and SDF1/CXCL12 (stromal cell-derived factor 1/C-X-C motif chemokine 12) from CAFs can drive cancer cell growth^14^,^15^. Moreover, the deposition of ECM components is integral to the ability of CAFs to generate a pro-invasive microenvironment. However, the complexity of CAF secretome renders it difficult to obtain a clear picture of how these cells contribute to cancer progression. Although a few studies have attempted to resolve the CAF secretome using mass spectrometry (MS)-based approaches, many of pro-invasive factors that are released by CAFs and the mechanisms through which they act remain unclear^16^,^17^. Using high-resolution MS we have comprehensively resolved the secretome of a validated model of human mammary CAFs^14^ and compared this with the secretome of normal mammary fibroblasts (NFs). We show that the CAF proteome gives insight into the capability of these cells to alter the extracellular environment and have elucidated protein components that indicate a new mechanism leading to a pro-invasive stroma in tumours. We show that the chloride intracellular channel protein 3 (CLIC3) is a prominent component of the CAF secretome and that this acts as a glutathione (GSH)-dependent oxidoreductase to influence the ability of secreted transglutaminase-2 (TGM2) to promote the invasive behaviour of both endothelial and cancer cells.

## Results

### The fibroblast secretome is altered upon activation into CAF

**Figure 1:**
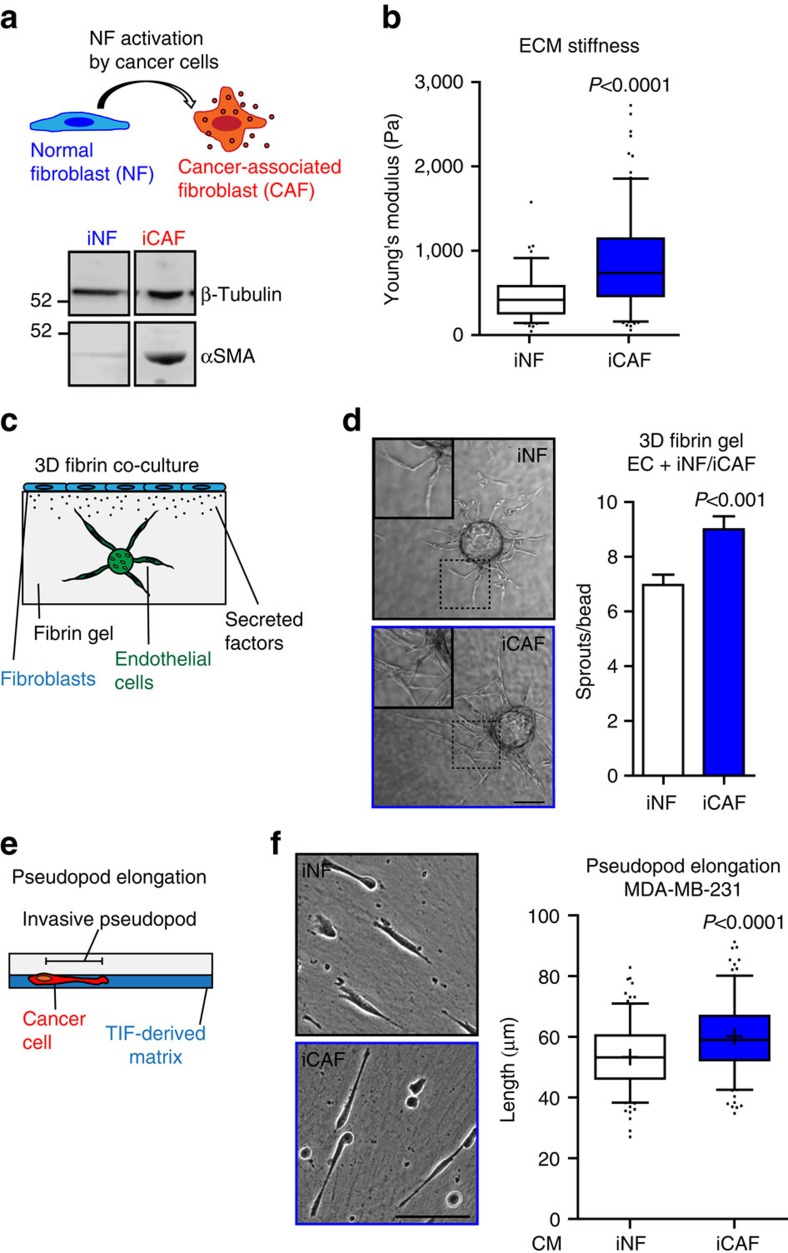
iCAFs generate stiff ECM and have a pro-invasive secretome. (**a**) Schematic explanation of the iNF-iCAF cell model. Below, the western blot shows the levels of the myofibroblast marker αSMA in total cell lysate of immortalized fibroblasts normal (iNF) and activated by MCF7-HRas cancer cells (iCAF). Vinculin was used as loading control. Full-size images of WB are presented in Supplementary Fig. 9. (**b**) Stiffness measurement of cell-free extracellular matrix (ECM). Whisker plot (10–90 percentile); *n*_iCAF_=179, *n*_iNF_=103 (*n*=fields assessed by AFM). (**c**) Cartoon representing the 3D fibrin gel co-culture system used to evaluate endothelial cell invasion. (**d**) Representative bright-field images (bar, 100 μm) and sprouting quantification of HUVEC co-cultured for 12 days with iNF and iCAF in 3D fibrin gel; *n*_iCAF_=31, *n*_iNF_=38 (*n*=HUVEC-coated beads assessed). (**e**) Cartoon representing the pseudopod elongation assay used to evaluate cancer cells invasion through telomerase immortalized fibroblast (TIF)-derived ECM. (**f**) Representative bright-field images (bar, 100 μm) and quantification of the invasive pseudopod length of MDA-MB-231 cells invading TIF-derived ECM in the presence of conditioned medium (CM) produced by iNF or iCAF. Whisker plot (5–95 percentile). The cross indicates the mean; *n*=540 cells assessed from 3 biological replicates. These samples are also reported in Fig. 4e. Bars, mean±s.e.m.

To elucidate the mechanisms that underpin the pro-invasive ability of fibroblasts upon activation into CAF by cancer cells, we used normal human mammary fibroblasts (iNF) and CAF (iCAF)^14^. These iCAFs were generated by serial passage of hTERT (human telomerase reverse transcriptase) immortalized normal human mammary fibroblasts through nude mice in the presence of HRas-transformed MCF7 breast cancer cells. The iNFs were obtained by similar passage through nude mice, but in the absence of cancer cells^14^. The iCAFs have a typical myofibroblast-like phenotype and express high levels of alpha-smooth muscle actin (αSMA)^18^ (Fig. 1a) and TGFβ that is maintained when grown in culture by positive feedback TGFβ signalling loop^14^. The iCAFs have greater capacity than iNFs to promote tumour vascularization and growth when co-injected with MCF7-HRas cells as subcutaneous xenograft^14^. First, we sought to evaluate the capability of iCAFs to directly affect the characteristics of the extracellular environment, and the invasive behaviour of endothelial cells (ECs) and cancer cells. Atomic force microscopy (AFM) analysis indicated that iCAFs produced a matrix that was significantly stiffer than that generated by iNFs (Fig. 1b). We used a three-dimensional (3D) culture system to investigate how factors released by fibroblasts may influence the invasiveness of ECs. We plated ECs into fibrin gels and overlayed these with either iNFs or iCAFs and monitored the invasive sprouting of the ECs in the gel (Fig. 1c). In the presence of iCAFs, the number of sprouts emanating from ECs was 25% greater than in the presence of iNFs (Fig. 1d). We also looked at the ability of conditioned medium (CM) from iCAFs to influence MDA-MB-231 breast cancer cells to extend invasive pseudopods into cell-free preparations of ECM (Fig. 1e). The CM from iCAFs was significantly more effective at promoting the extension of invasive pseudopods than was CM from iNFs (Fig. 1f). From this, we reasoned that the iNF-iCAF cell lines would be an excellent model to identify secreted factors necessary to generate a pro-invasive microenvironment.

**Figure 2:**
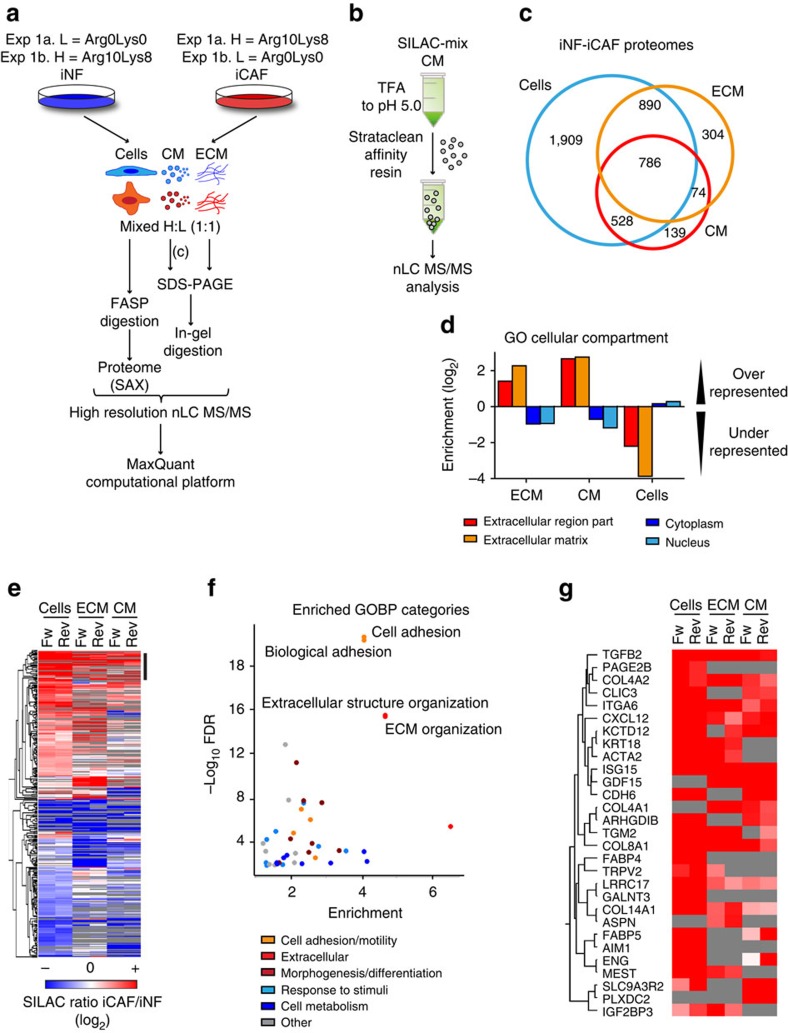
Proteomics and secretomics of iNF and iCAF. (**a**) MS-based approach workflow used for the comparative proteomic analysis of iNF and iCAF. Heavy SILAC-labelled iCAFs and light iNFs were used for the forward experiment (Exp 1a), and heavy SILAC-labelled iNFs and light iCAFs for the reverse experiment (Exp 1b). Cells, total cell proteome; CM, conditioned medium; ECM, extracellular matrix; FASP, filter-aided sample preparation (used for protein digestion); SAX, strong anion exchange (used to fractionate peptides). (**b**) Schematic workflow of the protocol developed to isolate proteins from serum-free CM produced by iNF and iCAF and used for SILAC-based proteomic quantification. TFA, trifluoracetic acid. Affinity resin, silica-based resin (Strataclean, Agilent Technologies). (**c**) Venn diagram of proteins quantified in both SILAC replicates by MS proteomics in cell, ECM and CM proteomes. (**d**) Fisher's test-based (2% Benjamini–Hochberg false discovery rate (FDR)) category enrichment analysis performed with Perseus on the indicated fractions, using the global proteome (cells, ECM and CM) as reference data set. Cells, proteins quantified in the cell but not in the CM and ECM proteomes; CM, proteins quantified in the CM but not in the cell proteome; ECM, proteins quantified in the ECM but not in the cell proteome. (**e**) Hierarchical clustering (based on average Euclidean distance) and heat map (colours based on SILAC ratio iCAF/iNF) of the SILAC ratio iCAF/iNF calculated for the 325 proteins of the iCAF signature. Cells, total cell lysate. (**f**) Gene ontology biological process (GOBP) enrichment analysis performed with STRING for the iCAF signature using the total proteome as background and Bonferroni test to correct for multiple testing. (**g**) Subcluster of most upregulated proteins in iCAFs (black line in (**e**)).

To accurately quantify proteomes, we used SILAC (stable isotope labelling by amino acids in cell culture)^19^ and compared the cellular, CM/secreted and ECM proteomes of iNFs and iCAFs. We prepared cell extracts and cell-free ECM using previously established protocols^20^,^21^, whereas for the CM we developed a simple affinity-based approach where Strataclean resin is used to enrich for proteins secreted into serum-free medium (Fig. 2a,b and Supplementary Fig. 1a). Proteomes were measured using an LTQ-Orbitrap mass spectrometer and MS data analysed with the MaxQuant computational platform^22^ that identified 5,467 proteins. Of these, 4,113 were reproducibly quantified in the cell proteome, 2,054 in the ECM and 1,527 in the CM (Fig. 2c, Supplementary Fig. 1b and Supplementary Data 1). The validity of our approach to isolate secreted proteins was demonstrated by Gene Ontology (GO) cellular compartment category enrichment analysis that indicated that proteins detected in the ECM and CM were significantly enriched in secretory proteins, whereas the subsets found within the cell extracts were enriched in cytoplasmic and nuclear proteins (Fig. 2d). Approximately 50% of the proteins identified in the CM and ECM fractions were also identified in the cell proteome (Fig. 2c). This group contained significant subsets of vesicles and extracellular organelle proteins (Supplementary Fig. 1c), suggesting that a substantial proportion of the fibroblast proteome may be secreted. A good correlation was measured between our data set and previously published breast cancer stroma signatures identified by gene expression analysis^23^,^24^,^25^ (Supplementary Fig. 2a–c and Supplementary Tables 1 and 2) that vindicates our use of the iNF and iCAF cell lines for this purpose. We assembled a signature of 325 proteins that were differentially regulated in iNFs and iCAFs (Fig. 2e, Supplementary Data 1 and Supplementary Methods). TGFβ signalling is the prominent pathway sustaining iCAF activation^14^ and, accordingly, our iCAF signature was characterized by increased levels of TGFβ-regulated proteins (Supplementary Fig. 3a). STRING analysis^26^ identified known physical and functional interactions between 164 proteins of the iCAF signature. The most highly connected network (Supplementary Fig. 3b) was enriched for extracellular and cell adhesion proteins (Fig. 2f). These included matrix components, such as collagen (COL)1A1, COL5A1, COL4A1, COL10A1, COL5A3, COL18A1, fibronectin (FN1), laminin (LAM) and Syndecan 2 (SDC2), and the matrix remodelling enzymes LOX and TGM2 that were found upregulated (Fig. 2g and Supplementary Data 1). Moreover, secreted factors (for example, thrombospondin 1 (THBS1) and biglycan (BGN)), and growth factors and cytokines (for example, TGFβ, CXCL12/SDF1, CTGF and FGF2), many of which actively control endothelial and cancer cell behaviour, were differentially regulated between iNFs and iCAFs (Fig. 2g and Supplementary Data 1). Thus, when fibroblasts become activated this is particularly noticeable in alterations to their secretome and the ECM proteins deposited by these cells. Our iCAF signature reveals the presence of a number of components that are potential novel regulators of angiogenesis and tumour progression.

### CLIC3 is secreted by CAFs and cancer cells

**Figure 3:**
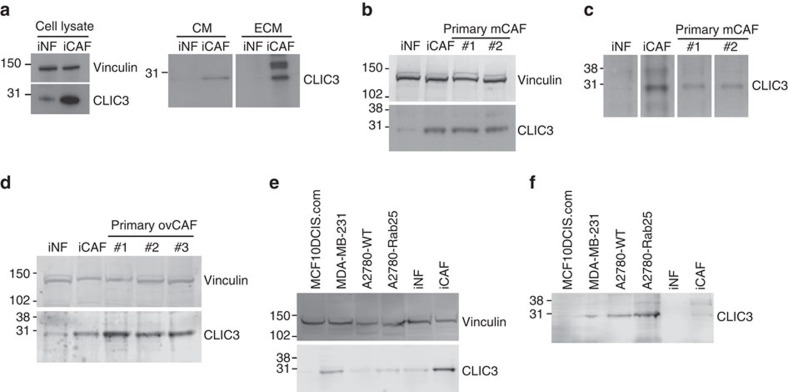
CLIC3 is abundant in activated fibroblasts and deposited in the ECM by CAF and cancer cells. (**a**) Western blot for CLIC3 in iNF and iCAF cell lysate, conditioned medium (CM) and extracellular matrix (ECM). Vinculin was used as loading control for the cell lysate. (**b**,**c**) Western blot for CLIC3 in cell lysate (**b**) and ECM (**c**) of cultured primary mammary CAF (mCAF) isolated from tumour tissue of two breast cancer patients. The iNF and iCAF cell lysates were loaded for comparison. Vinculin was used as loading control. In **c**, the brightness of the panels has been increased using Photoshop. (**d**) Western blot for CLIC3 in cell lysate of cultured primary ovarian CAF (ovCAF) isolated from tumour-omentum (#1, #2) and tumour tissue (#3) of ovarian cancer patients. The iNF and iCAF cell lysates were loaded for comparison. Vinculin was used as loading control. (**e**,**f**) Western blot for CLIC3 in the total lysate (**e**) and ECM (**f**) of the indicated cancer cells showing that MCF10DCIS.com express very low levels of CLIC3 and that MDA-MB-231 breast cancer cells and A2780 cells can deposit CLIC3 in the ECM, as shown for iCAFs. A2780-Rab25, A2780 cells stably expressing Rab25 that has been shown to increase CLIC3 levels^31^. Vinculin was used as loading control for the cell lysate. Full-size images of WB are presented in Supplementary Figs 9 and 10.

Among the proteins not found in the interaction network and which were most upregulated in the iCAF proteome and secretome (Fig. 2g), we were intrigued by CLIC3. A function for CLIC3 in fibroblasts is unknown, and the observation that it was found in the extracellular milieu suggested previously unexplored functions for this protein. CLIC3 belongs to a family of proteins mostly described as intracellular Cl^−^ channels and scaffolding proteins^27^,^28^,^29^. We have previously shown that CLIC3 localizes in the late endosomal compartment of cancer cells and determines cell invasion and metastasis by promoting the trafficking of α_5_β_1_ integrin and the transmembrane matrix metalloprotease MT1-MMP to the plasma membrane^30^,^31^. Intriguingly, there is increasing evidence that CLIC proteins are secreted and detected in body fluids^32^,^33^, but an extracellular role for the CLICs has not been reported. Western blotting confirmed that CLIC3 was upregulated in iCAFs and showed that it was released into the medium and incorporated into the ECM (Fig. 3a). Similar levels of CLIC3 were measured in primary mammary CAFs and iCAFs (Fig. 3b), and detected in the ECM deposited by primary mammary CAFs (Fig. 3c). Analysis of publicly available gene expression data sets indicated higher CLIC3 levels in the stroma of ovarian (GSE40595), oral (GEOD-38517) and colon (GSE35602) carcinoma when compared with stroma of the corresponding normal tissues (Supplementary Table 3). We confirmed the expression of CLIC3 in primary ovarian CAFs isolated from patient samples by western blot (Fig. 3d). Hence, CLIC3 expression is enhanced in the stroma of different tumours. We have previously shown that CLIC3 levels are high in cancer cells of aggressive cancers^30^,^31^. Here we determined that cancer cells also secrete CLIC3. Indeed, western blot analysis detected CLIC3 in the ECM produced by CLIC3-expressing MDA-MB-231 and A2780 cancer cells, but not MCF10DCIS.com cells that have low levels of CLIC3 (Figs 3e,f). Thus, we have identified CLIC3 as a factor that can be released extracellularly by activated fibroblasts and cancer cells.

### CLIC3 promotes cell invasion

**Figure 4:**
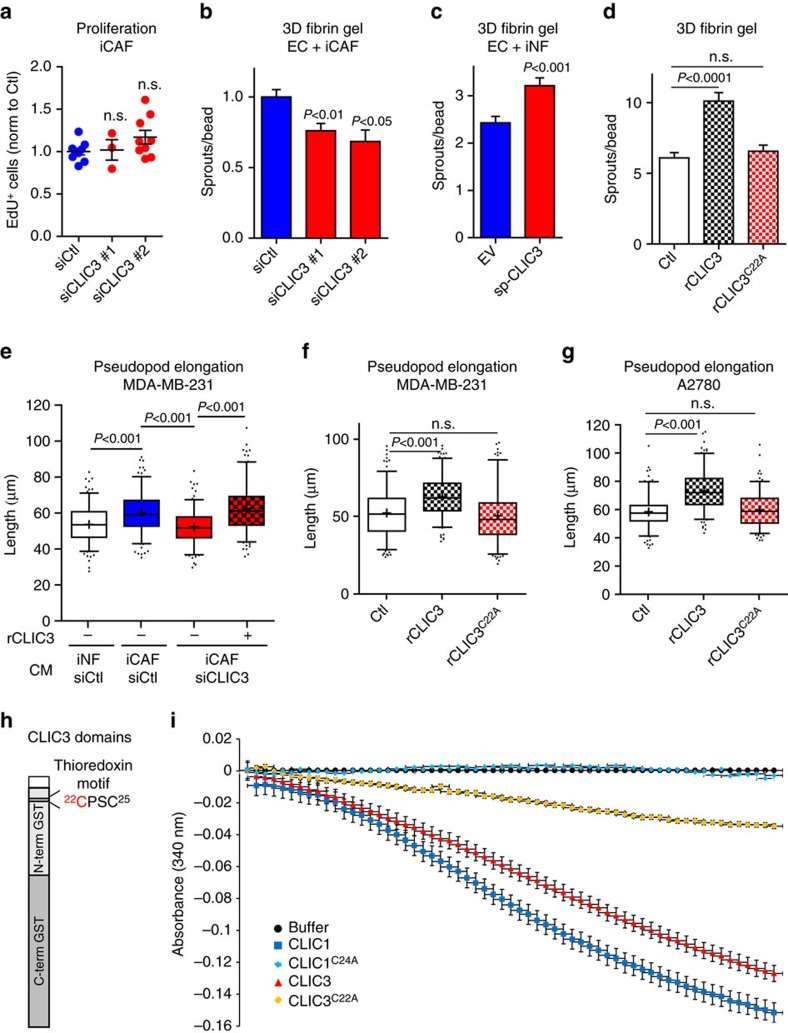
CLIC3 is a glutathione-dependent oxidoreductase enzyme. (**a**) Proliferation of iCAF silenced for CLIC3 with two independent siRNAs, measured by means of % of cells that incorporated EdU (=cells in S phase). The results shown are from three biological replicates for siCtl and siCLIC3 #1 and two for siCLIC3 #2. (**b**) Sprouting quantification of HUVECs co-cultured in 3D fibrin gel for 6 days with siCtl or siCLIC3 iCAF. Each siCLIC3 was normalized by and statistics calculated based on its siCtl; *n*_siCtl_=66, *n*_siCLIC3#1_=48, *n*_siCLIC3#2_=32 (*n*=HUVEC-coated beads assessed). (**c**) Sprouting quantification of HUVECs co-cultured in 3D fibrin gel for 4 days with iNFs stably overexpressing a modified secreted form of CLIC3 containing a signal peptide (spCLIC3) or the control empty vector (EV); *n*_EV_=89, *n*_spCLIC3_=85 (*n*=HUVEC-coated beads assessed). (**d**) Sprouting quantification of HUVECs in 3D fibrin gel stimulated for 2 days with vehicle (Ctl), rCLIC3 (250 ng ml^−1^) or rCLIC3^C22A^ (250 ng ml^−1^); *n*_siCtl_=151, *n*_rCLIC3_=135, *n*_rCLIC3C22A_=145 (*n*=HUVEC-coated beads assessed from three biological replicates). (**e**) Quantification of invasive pseudopod length of MDA-MB-231 cells migrating on cell-free ECM produced by telomerase immortalized fibroblasts (TIFs) and in the presence of conditioned medium (CM) generated by siCtl iCAF or iNF or CLIC3-silenced iCAF. rCLIC3=1 ng ml^−1^. Whisker plot (5–95 percentile). The cross indicates the mean; *n*=540 (for all but *n*_siCLIC3+rCLIC3_=500) cells assessed from 3 biological replicates. iNF and iCAF siCtl are the same reported in Fig. 1f. (**f**,**g**) Quantification of invasive pseudopod length of MDA-MB-231 breast (**f**) and A2780 ovarian (**g**) cancer cells migrating on cell-free ECM produced by TIFs and treated with GST (25 ng ml^−1^, Ctl), rCLIC3 (25 ng ml^−1^) or rCLIC3^C22A^ (25 ng ml^−1^). Whisker plot (5–95 percentile). The cross indicates the mean. For MDA-MB-231 cells, *n*_Ctl_=360, *n*_rCLIC3_=720, *n*_rCLIC3C22A_=330 cells assessed from two biological replicates; for A2780 cells *n*_Ctl_=480, *n*_rCLIC3_ and *n*_rCLIC3C22A_=540 cells assessed from three biological replicates. (**h**) Scheme of CLIC3 domains highlighting the N-terminal thioredoxin motif. In red, the cysteine conserved among CLICs and which we have shown to be enzymatically active (**i**). (**i**) Enzymatic assay showing the glutathione-dependent oxidoreductase activity of CLIC1 and CLIC3 wild type or when mutated at the active cysteine (cysteine 24 for CLIC1, CLIC1^C24A^; cysteine 22 for CLIC3, CLIC3^C22A^). Buffer only was used as negative control for the enzymatic reaction. Bars indicate average±s.d.; *n*=3 technical replicates. Bars, mean±s.e.m.

Despite clear indications that CLIC3 is not required for proliferation of iCAFs (Fig. 4a and Supplementary Fig. 4a), CLIC3 knockdown (with two independent small interfering RNAs (siRNAs)) reduced the ability of iCAFs (Fig. 4b and Supplementary Fig. 4b,c) and primary mammary CAFs (Supplementary Fig. 4d,e) to promote EC sprouting in 3D fibrin gel. This was likely because of defects in EC invasiveness and not proliferation because treatment of ECs with CM from iCAFs silenced for CLIC3 had no significant impact on EC proliferation (Supplementary Fig. 4f). Given our MS data indicating that CLIC3 is secreted, we tested whether CLIC3 influence on ECs was mediated extracellularly. We stably overexpressed a secreted signal peptide-containing form of CLIC3 (spCLIC3) in iNFs (Supplementary Fig. 4g). Strikingly, compared with iNFs expressing an empty control vector, iNFs-spCLIC3 enhanced EC sprouting (Fig. 4c), indicating that extracellular CLIC3 is biologically active. Further evidence of the extracellular role of CLIC3 came from experiments in which we measured the effect of soluble purified recombinant CLIC3 (rCLIC3) on EC sprouting, when co-cultured with iNFs. Addition of purified rCLIC3 to the medium promoted EC sprouting in a dose-dependent manner (Supplementary Fig. 4h). Importantly, rCLIC3 drove EC sprouting in fibrin gels in the absence of a fibroblast overlay (Fig. 4d), and enhanced vascular endothelial growth factor (VEGF)-driven EC sprouting from mouse aortic rings explanted into Matrigel (Supplementary Fig. 4i). To determine whether secreted CLIC3 contributed to cancer cell invasiveness, we incubated MDA-MB-231 cells with conditioned medium from iCAFs in which CLIC3 had been knocked down (Supplementary Fig. 5a). The CM from CLIC3-knockdown iCAFs had reduced ability to drive the extension of invasive pseudopods from MDA-MB-231 cells, and this was completely restored by replacement of CLIC3 in the iCAF-CM with rCLIC3 (Fig. 4e). Moreover, addition of rCLIC3 was sufficient to drive extension of invasive pseudopods from both MDA-MB-231 breast and A2780 ovarian cancer cells in a dose-dependent manner, even in the absence of CM from iCAFs (Fig. 4f,g and Supplementary Fig. 5b,c). Consistently, addition of rCLIC3 was sufficient to increase the invasiveness of A2780 cells into Matrigel plugs^34^ (Supplementary Fig. 5d). Taken together, these data indicate that the extracellular pool of CLIC3 is necessary and sufficient to drive the invasiveness of both ECs and cancer cells. Next, we investigate the mechanisms of CLIC3 function.

### CLIC3 is a GSH-dependent oxidoreductase enzyme

It has recently been shown that some CLIC proteins have GSH-dependent oxidoreductase activity^29^. We used an assay that measures the reduction of bis(2-hydroxyethyl)disulfide (HEDS) to establish that rCLIC3 possessed GSH-dependent oxidoreductase activity that was comparable to that of CLIC1. The glutaredoxin-like activity of CLIC1 requires the presence of a conserved cysteine in its N-terminal thioredoxin-like domain^29^. We mutated the corresponding cysteine residue in CLIC3 to generate rCLIC3^C22A^ (Fig. 4h and Supplementary Fig. 5e–g). As expected, this mutant had strongly reduced GSH-dependent oxidoreductase activity (Fig. 4i). To determine whether CLIC3 oxidoreductase activity may contribute to its capacity to drive invasiveness, we tested the ability of CLIC3^C22A^ to drive EC sprouting and extension of invasive pseudopods from tumour cells. It was clear that rCLIC3^C22A^ had significantly reduced capacity to drive the invasiveness of endothelial, MDA-MB-231 and A2780 cells (Fig. 4d,f,g). Hence, the oxidoreductase activity of CLIC3 may contribute to the pro-invasive role of extracellular CLIC3.

### CLIC3 reduces TGM2 and alters TGM2 binding with its cofactors

**Figure 5:**
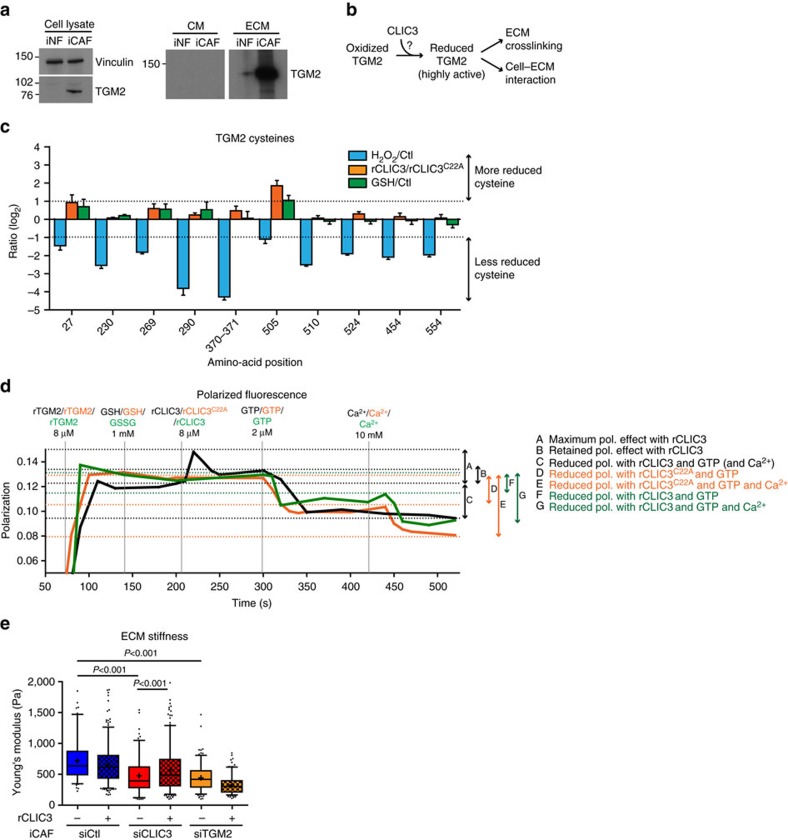
CLIC3 reduces TGM2 and controls the binding between TGM2 and its cofactors. (**a**) Western blot for TGM2 of iNF and iCAF total cell lysate, conditioned medium (CM) and extracellular matrix (ECM). Vinculin (same image as in Fig. 3b) was used as loading control. Full-size images of WB are presented in Supplementary Fig. 9. (**b**) Scheme showing the possible cooperation between extracellular CLIC3 and TGM2. (**c**) Ratio of reduced cysteine levels between the indicated samples, as measured by MS. On the *x* axis are the cysteines that were found reduced in TGM2 and that are susceptible to oxidation upon H_2_O_2_ treatment (10–100 μM). rCLIC3/rCLIC3^C22A^=both reactions were performed in the presence of GSH; GSH/Ctl=both reactions were performed using rCLIC3. Dashed bars indicate a twofold difference in reduced cysteine levels between samples (*n*=4 MS measurements from two independent experiments). (**d**) Polarization (pol.) fluorescence measurements using 2 μM Mant-GMPPNP followed by addition of rTGM2, GSH (black, orange) or GSSG (green), rCLIC3 (black) or rCLIC3^C22A^ (orange) as indicated in the figure. Unlabelled GTP (GTP) and Ca^2+^ were added to displace the Mant-GMPPNP. The *y* axis starts at 0.05 and full range is reported in Supplementary Fig. 6c. (**e**) Stiffness measurement by atomic force microscopy of cell-free ECM produced by siCtl, siCLIC3 or siTGM2 iCAF grown in the presence of recombinant GST (Ctl, 250 ng ml^−1^) or recombinant CLIC3 (rCLIC3, 250 ng ml^−1^). Whisker plot (10–90 percentile); *n*_Ctl_=106, *n*_Ctl-CLIC3_=288, *n*_siCLIC3_=151, *n*_siCLIC3-CLIC3_=276, *n*_siTGM2_=180, *n*_TGM2-CLIC3_=183 (*n*=fields assessed by AFM). Data are representative of two independent experiments. Bars, mean±s.e.m.

TGM2 was among the most upregulated proteins of the iCAF secretome (Figs 2g and 5a). TGM2 can crosslink and stabilize the ECM^35^, and it controls cell–matrix interactions by binding to membrane receptors such as β1 integrin^36^. Both matrix stiffness and integrin activation promote cell invasion^5^,^6^. Moreover, TGM2 activity is controlled by a thioredoxin-mediated reduction of cysteines 370–371 (refs 37, 38). For these reasons, we considered the possibility that CLIC3 worked in collaboration with TGM2 (Fig. 5b). We determined that CLIC3 controls the reduced status of TGM2 cysteines by using quantitative MS (see Supplementary Methods). First, H_2_O_2_ treatment of GTP-bound recombinant TGM2 (rTGM2) showed that the cysteines at positions 10, 27, 230, 269, 370–371, 505, 510, 524 and 554 can be reduced and susceptible to oxidation (Fig. 5c). Next, we incubated GTP-bound rTGM2 with rCLIC3 or rCLIC3^C22A^ and compared the reduced status of TGM2 cysteines by MS. This analysis revealed that cysteine 505 was fivefold more reduced when TGM2 was incubated with the enzymatically active rCLIC3 compared with the inactive mutant rCLIC3^C22A^ (Fig. 5c). Similar results were obtained when TGM2 was incubated with rCLIC3 in the presence or absence of reduced GSH (Fig. 5c). Higher reduction levels (1.5–2-fold) were also measured for cysteines 27, 269 and 370–71 when TGM2 was incubated with rCLIC3 compared with when incubated with rCLIC3^C22A^ (Fig. 5c). These data indicate that CLIC3 controls TGM2 reduction at specific cysteines.

We next wished to determine whether CLIC3 and TGM2 interaction influenced TGM2 activity. We used a fluorescence polarization assay that exploited the fact that TGM2 is a GTP/GDP-binding protein. First we confirmed the capability of recombinant TGM2 to bind to the fluorescently labelled non-hydrolyzable GTP analogue, Mant-GMPPNP, and that this interaction is disrupted in the presence of free Ca^2+^, as reported previously (Supplementary Fig. 6a). Then, we explored CLIC3 interaction with TGM2. Starting with Mant-GMPPNP, we observed incremental increases in the polarization signal upon step-wise addition of TGM2 followed by rCLIC3, but not rCLIC3^C22A^ (Fig. 5d and Supplementary Fig. 6b). This increase was observed in the presence of reduced GSH, but not its oxidized form (Fig. 5d and Supplementary Fig. 6b). As TGM2 was at subsaturating concentration, the increase in the polarization signal could indicate either that rCLIC3 physically interacts with Mant-GMPPNP.TGM2 or that it regulates the affinity between TGM2 and Mant-GMPPNP. Strikingly, we noticed that rCLIC3, but not rCLIC3^C22A^, prevented Ca^2+^-induced release of Mant-GMPPNP (Fig. 5d), indicating that CLIC3 regulates the binding of TGM2 to Ca^2+^ and GTP that are the cofactors regulating TGM2 activities.

Finally, we determined that TGM2 is required for the function of extracellular CLIC3. The iCAFs silenced for CLIC3 (Supplementary Fig. 6c) generated an ECM with significantly reduced stiffness, and this was almost completely restored simply by adding rCLIC3 to the culture medium of iCAF silenced for CLIC3 (Fig. 5e). Conversely, the addition of rCLIC3 to iCAF expressing endogenous levels of CLIC3 was not able to induce a further increase of matrix stiffness (Fig. 5e). This suggests that endogenous levels of CLIC3 were sufficient to stiffen the iCAF ECM and that iCAFs cannot generate a stiffer ECM. Conversely, when iCAFs were silenced for TGM2 (Supplementary Fig. 6c), they generated a matrix with significantly reduced stiffness, but this was not restored by adding rCLIC3 (Fig. 5e).

### CLIC3 requires TGM2 to promote cell invasion

**Figure 6:**
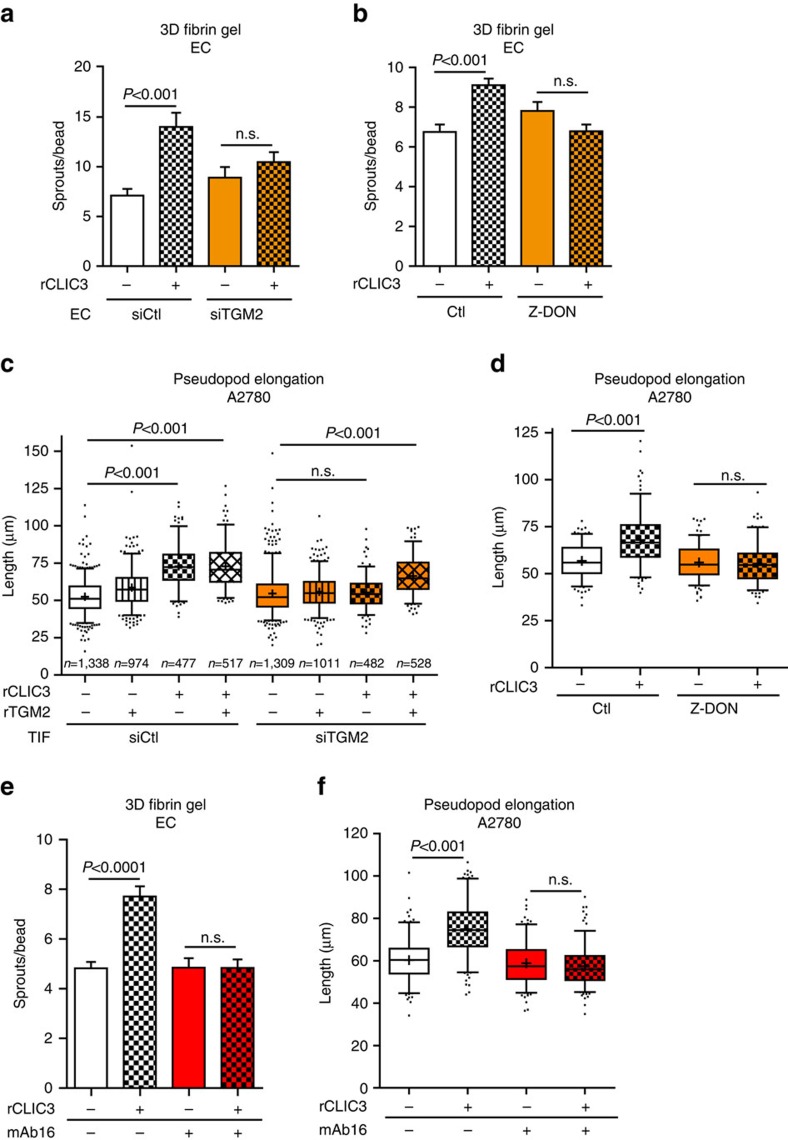
Extracellular CLIC3 requires TGM2 to be pro-invasive *in vivo* and 3D models *in vitro*. (**a**) Sprout number quantification of HUVEC silenced for TGM2 (siTGM2) or nontargeting siRNA (siCtl) and cultured in 3D fibrin gel and treated with vehicle (Ctl) or rCLIC3 (250 ng ml^−1^) for 2 days; *n*_Ctl_=27, *n*_Ctl-CLIC3_=20, *n*_siTGM2_=22, *n*_TGM2-CLIC3_=21 (*n*=HUVEC-coated beads assessed). (**b**) Sprout number quantification of HUVEC cultured in 3D fibrin gel and treated or not with rCLIC3 (250 ng ml^−1^) in the presence or absence of Z-DON (20 nM) to inhibit TGM2; *n*_Ctl_=48, *n*_Ctl-CLIC3_=49, *n*_Z-DON_=43, *n*_ZDON-CLIC3_=37 (*n*=HUVEC-coated beads assessed). (**c**) Quantification of invasive pseudopod length of A2780 cells migrating on cell-free ECM produced by siCtl or siTGM2 telomerase immortalized fibroblasts (TIFs) and treated with GST (1 ng ml^−1^), rTGM2 (0.9 ng ml^−1^), rCLIC3 (1 ng ml^−1^) or in combination. The cross indicates the mean. Whisker plot (5–95 percentile); *n* (=cells assessed from three biological replicates) is indicated in the figure. (**d**) Quantification of invasive pseudopod length of A2780 cells migrating on ECM produced by siCtl or siTGM2 TIFs and treated with GST (1 ng ml^−1^) or rCLIC3 (1 ng ml^−1^) in the presence or absence of Z-DON (20 nM). Whisker plot (5–95 percentile). The cross indicates the mean; *n*=540 (*n*=cells assessed from three biological replicates). (**e**) Sprout number quantification of HUVEC cultured in 3D fibrin gel and treated or not with rCLIC3 (250 ng ml^−1^) in the presence or absence of mAb16 (20 nM) to inhibit α5 integrin; *n*_Ctl_=57, *n*_Ctl-CLIC3_=42, *n*_mAb16_=46, *n*_mAb16 -CLIC3_=36 (*n*=HUVEC-coated beads). (**f**) Quantification of invasive pseudopod length of A2780 cells migrating on ECM produced by TIFs and treated with GST (1 ng ml^−1^) or rCLIC3 (1 ng ml^−1^) in the presence or absence of mAb16 (20 nM). Whisker plot (5–95 percentile). The cross indicates the mean; *n*=540 (*n*=cells assessed from three biological replicates). Bars, mean±s.e.m.

Next, we investigated the requirement of TGM2 in extracellular CLIC3 ability to promote invasion. The ability of rCLIC3 to drive EC sprouting was TGM2 dependent. In fact, rCLIC3-driven EC sprouting was completely opposed by knockdown of TGM2 in ECs (Fig. 6a and Supplementary Fig. 7a). Moreover, a well-characterized inhibitor of TGM2 activity, Z-DON^39^, blocked rCLIC3-driven EC sprouting when used at a concentration that did not affect cell viability and that would not be expected to penetrate the cell^39^ (Fig. 6b and Supplementary Fig. 7b,c). As rCLIC3-induced but not VEGF-induced EC sprouting was inhibited by Z-DON (Supplementary Fig. 7d), we conclude that the effect of TGM2 blockade was specific for CLIC3-induced sprouting.

Next, we assessed the requirement of TGM2 in extracellular CLIC3 ability to promote cancer cell invasion. The ability of rCLIC3 to drive pseudopod elongation was completely ablated when cancer cells were plated into ECM generated by TGM2 knockdown fibroblasts (Fig. 6c and Supplementary Fig. 7e). Strikingly, addition of purified active rTGM2 to the culture medium was sufficient to restore rCLIC3-induced pseudopod elongation (Fig. 6c). In addition, in the presence of Z-DON, rCLIC3-driven pseudopod elongation was completely opposed (Fig. 6d). Similar results were obtained using a 3D Matrigel-based invasion assay (Supplementary Fig. 5d).

Finally, we determined that α_5_β_1_ integrin, of which the β1 subunit can be bound and activated by the GTP-bound TGM2 (ref. 36), was required for CLIC3-induced invasion. Indeed, the ability of CLIC3 to drive endothelial (Fig. 6e) and cancer cell (Fig. 6f) invasion was blocked in the presence of function-blocking antibody that recognizes the α5 subunit of α_5_β_1_ integrin^40^.

Taken together, these data indicate that secreted CLIC3 requires the activity of extracellular TGM2 and α_5_β_1_ integrin to promote the invasive behaviour of both ECs and cancer cells.

### CLIC3 drives angiogenesis and tumour invasion

**Figure 7:**
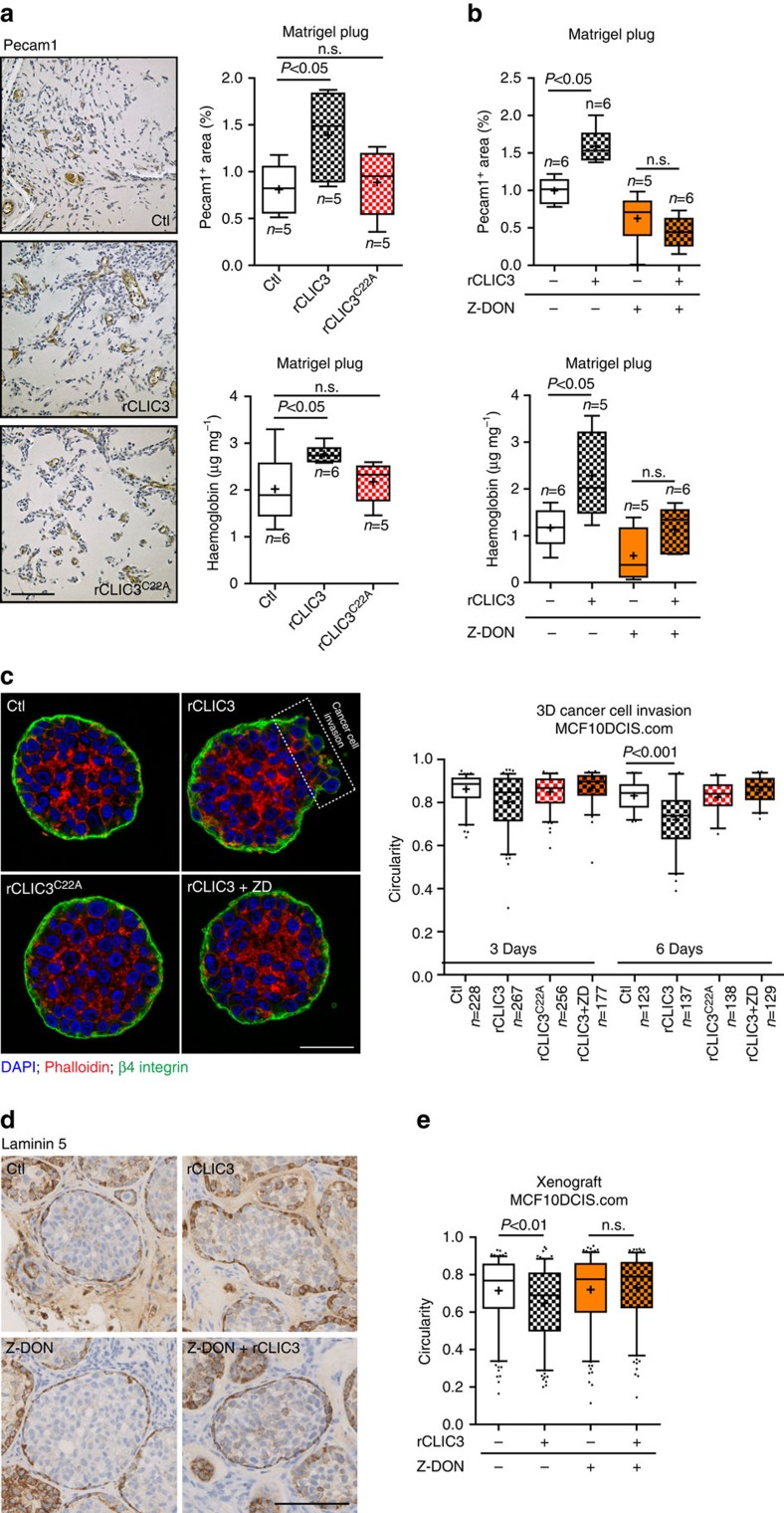
Extracellular CLIC3 is pro-invasive *in vivo* and 3D models *in vitro*. (**a**) Representative immunohistochemistry staining for the endothelial marker Pecam1 and quantification of functional blood vessels, by means of area (%) containing positive staining for Pecam1 (above) or amount of haemoglobin (below), of FGF2-containing Matrigel plugs embedded with vehicle (Ctl) or rCLIC3/rCLIC3^C22A^ (33 μM) that were implanted subcutaneous in mice for 10 days; *n* (*n*=mice) is indicated in the figure. Scale bar, 100 μm. (**b**) Quantification of the functional vascularity of FGF2-containing Matrigel plugs containing vehicle or rCLIC3 (33 μM) with or without Z-DON (2 μM) that were implanted subcutaneous in mice for 10 days; *n* (*n*=mice) is indicated in the figure. (**c**) Representative immunofluorescence staining for β4 integrin and actin (phalloidin) and quantification of the circularity of comedo-like DCIS structure formed by iMCF10DCIS.com cells cultured for 3 and 6 days in Matrigel, which formed in the presence of GST (Ctl, 25 ng ml^−1^) or rCLIC3/rCLIC3^C22A^ (25 ng ml^−1^), in the presence or absence of Z-DON (20 nM, ZD). Whisker plot (5–95 percentile). Bar, 40 μm; *n* (*n*=comedo-like DCIS structure assessed from three biological replicates) is indicated in the figure. The dashed box highlights region where the cancer cells have breached the basal membrane and invaded the Matrigel. (**d**) Representative laminin 5 immunohistochemistry and quantification of noninvasive tumour formed by MCF10DCIS.com cells grown for 2 weeks as subcutaneous xenograft in the presence of vehicle or rCLIC3 (33 μM) in the presence or absence of Z-DON (20 nM). Bar, 100 μm. (**e**) Quantification of the circularity of DCIS-like structures formed by MCF10DCIS.com cells grown for 2 weeks as subcutaneous xenograft in the presence of vehicle or rCLIC3 (33 μM) in the presence or absence of Z-DON (20 nM). Bar, 100 μm. *N*=180 DCIS-like structures/group (=30 DCIS-like structures assessed/mouse/group). In all the plots, the cross indicates the mean.

We asked whether extracellular CLIC3 generated pro-invasive outcomes in *in vivo* and 3D models. To determine the influence of extracellular CLIC3 on EC behaviour *in vivo*, Matrigel plugs impregnated with FGF2 in the presence and absence of CLIC3 were subcutaneously implanted into mice. By immunohistochemical staining for Pecam1 to visualize endothelial cells and haemoglobin measurements to verify blood vessel functionality, we found that addition of rCLIC3, but not rCLIC3^C22A^, significantly increased functional vascularization of the plug (Fig. 7a). Furthermore, we inhibited TGM2 with the inclusion of Z-DON within the plug. When this inhibitor was included in the plug together with rCLIC3, rCLIC3 was unable to significantly enhance angiogenesis (Fig. 7b). Next, to investigate cancer cell invasion, we deployed MCF10DCIS.com breast cancer cells. This model has the advantage that both *in vitro* and *in vivo* assays recapitulates the progression from DCIS to invasive carcinoma^41^,^42^. When cultured in Matrigel for up to 5 days, MCF10DCIS.com cells formed noninvasive comedo-like spheres bounded by basement membrane (BM), as determined by immunofluorescence staining for laminin 5 and β4 integrin (Fig. 7c and Supplementary Fig. 7f). Addition of rCLIC3 to the extracellular milieu did not influence the initial assembly of DCIS structures, but the tumour spheres displayed loss of sphericity after 6 days of culture with rCLIC3, but not rCLIC3^C22A^ (Fig. 7c). The loss of sphericity was associated with a pronounced disruption of the BM, a well-established indicator of cancer cell invasion^30^. Moreover, the ability of CLIC3 to accelerate BM disruption was completely blocked by addition of Z-DON (Fig. 7c). Finally, to mimic an excess of extracellular CLIC3 in the tumour microenvironment, regardless of its origin (stromal and/or cancer cells), we mixed MCF10DCIS.com cells with Matrigel in the presence and absence of rCLIC3 and injected this subcutaneously into mice. Immunohistochemical staining showed that MCF10DCIS.com cells injected in the absence of CLIC3 formed numerous DCIS-like structures bounded by a laminin 5-containing basement membrane (Fig. 7d). In the presence of extracellular CLIC3, tumours had a more invasive phenotype, as assessed by the presence of DCIS-like structures with reduced sphericity (spherical related to *in situ*; nonspherical related to invasive) (Fig. 7d,e). Strikingly, when Z-DON was added into the Matrigel to inhibit TGM2, rCLIC3 was incapable of significantly enhancing invasion (Fig. 7d,e). Hence, the CLIC3/TGM2 pathway promotes angiogenesis *in vivo*, and breast cancer invasion in 3D culture and *in vivo*.

### High CLIC3 levels in ovarian tumours indicate poor outcome

Analysis of tissue microarrays (TMAs) that we have previously stained for CLIC3 (refs 30, 31) indicated that 90% of ovarian, 20% of breast and almost none of the pancreatic cancers stained positively for CLIC3 in the stroma. As in ovarian tumours CLIC3 was detectable both in the stroma and cancer cells in the vast majority of the patient samples, we chose this cancer type to investigate the relationship between CLIC3 levels in cancer cells and stromal compartment, and whether CLIC3 levels associated with a clinical outcome.

**Figure 8:**
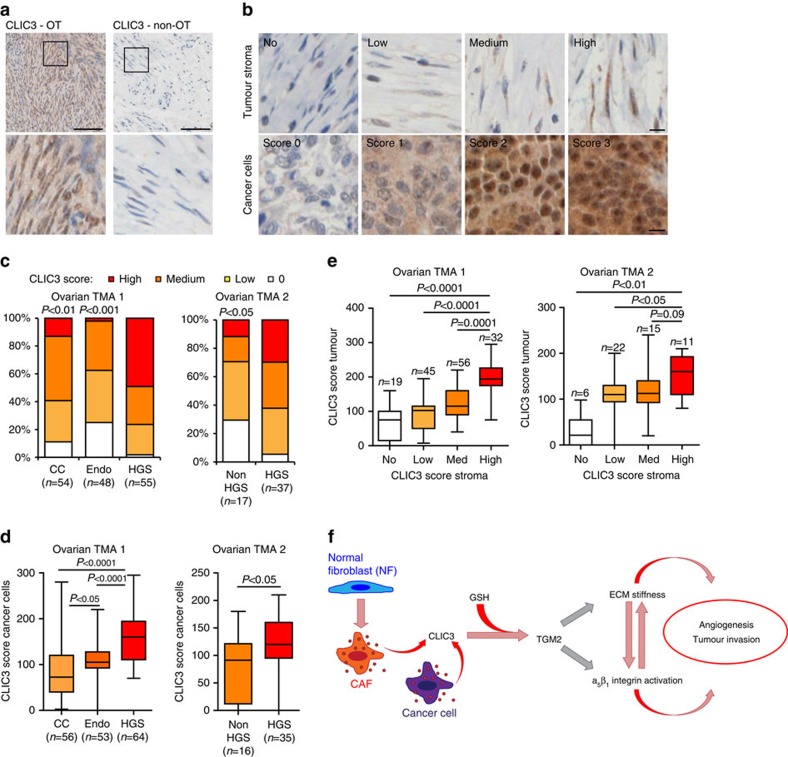
High CLIC3 levels associates with ovarian cancer clinical outcome. (**a**) Example of positive and negative immunohistochemistry staining for CLIC3 in the stroma of ovarian tumour (OT) and normal (non-OT, uterus) samples, respectively. Bar, 100 μm. (**b**) Example of immunohistochemistry staining used to measure CLIC3 histoscore in the stroma and cancer cells of ovarian tumours. Bar, 10 μm. (**c**) Stacked bars showing the prevalence of high and medium CLIC3 histoscore in the stroma of HGS compared with clear cell (CC) and endometrioid (Endo) ovarian cancers in two cohorts of patients (TMA 1 and TMA 2); CC and Endo were pooled together as ‘non-HGS' in TMA 2 to increase the number of samples for comparison with HGS. (**d**) Whisker plot (min to max) showing the higher CLIC3 histoscore in the cancer cells of HGS compared with clear cell and endometrioid ovarian cancers in two cohorts of patients (TMA 1 and TMA 2). (**e**) Whisker plot (min to max) that shows similar trend of CLIC3 histoscore in the cancer cell and stroma of the ovarian cancer TMA 1 and 2; *n*=number of single core assessed. (**f**) Working model for CLIC3-TGM2 cooperation to drive angiogenesis and tumour invasion. Grey arrows indicate the detailed mechanism through which CLIC3-activated TGM2 drives ECM stiffness and possibly integrin activation has still to be determined. The number of cores assessed (*n*) is indicated in the plot. Of the assessed cores, there were 2–4 cores/patient/tumour type.

Immunohistochemistry staining indicated that CLIC3 was expressed in the stroma of ovarian tumours but not in the stroma of the corresponding normal tissue (uterus) (Fig. 8a). Moreover, CLIC3 was detected in the tumour stroma that also stained positively for αSMA and TGM2 (Supplementary Fig. 8a). CLIC3 levels in the cancer cells and stroma of two independent TMAs were measured by immunostaining. Histoscore of CLIC3 staining in the stroma (Fig. 8b) showed higher CLIC3 levels in high-grade serous (HGS) compared with less aggressive^43^ clear cell and endometrioid cancer subtypes (Fig. 8c). Similarly, CLIC3 levels in cancer cells were higher in more aggressive HGS cancers (Fig. 8d), in line with our previous findings^30^,^31^. Clearly, CLIC3 levels were similarly regulated in stroma and cancer cells (Fig. 8e). Highlighting the fact that CLIC3 levels may be relevant to clinical outcomes, overall survival (OS) in HGS patients was lower when high (average OS=16.4 months in TMA 1 and 4 months in TMA 2) compared with low/medium (average OS=24.7 months in TMA 1 and 9.8 months in TMA 2) stromal CLIC3 histoscore was measured (Table 1). Concordantly, HGS patients with higher levels of CLIC3 in cancer cells had poorer survival (Table 1). Corroborating our findings, in a study conducted by Gyorffy *et al*.^44^, patients with HGS ovarian cancer with high CLIC3 mRNA levels in the tumour had reduced overall survival compared with those with low CLIC3 (Supplementary Fig. 8b). Taken together, these data indicate that high CLIC3 levels in aggressive ovarian cancers are associated with poor patient outcomes.

## Discussion

The contribution made by the microenvironment to tumour progression is underpinned by autocrine and paracrine signalling in which the secretome of CAFs and cancer cells plays a pivotal role. Here we show that modern mass spectrometry and SILAC for robust protein quantification combined with a protocol that we have developed to easily access secreted proteins in culture cells is a powerful approach for the unbiased discovery of invasive pathways in cancer.

A facet of the CAF secretome that is well established to influence tumour progression is the TGFβ-related network, and the iCAF model that we have deployed has previously been key to the identification of these pathways^14^. Rapidly accumulating evidence supports a role for ECM remodelling enzymes in promoting cancer cell invasion and angiogenesis^6^,^45^. TGM2 may also participate in these processes. In fact, when bound to Ca^2+^, TGM2 stiffens the ECM and couples integrins to fibronectin by generating covalent crosslinks between lysine and glutamine residues of adjacent peptide chains. When bound to GTP, TGM2 activates integrin by directly binding to the β1 subunit^36^,^46^. Here we have identified a new pathway whereby extracellular CLIC3 cooperates with TGM2 to drive endothelial and cancer cell invasion.

Members of the CLIC family are abundant in tumour and stromal compartments. CLIC4 influences TGFβ signalling in tumour cells to promote cell growth^47^ and CLIC3 drives integrin and metalloproteinase recycling to increase invasiveness^30^,^31^. However, the way in which CLICs influence cancer progression through altering the tumour microenvironment is not yet clear. As previously shown for CLIC4 (ref. 48), CLIC3 may act within stromal cells to influence TGFβ signalling, but our data indicate that it is the extracellular pool of CLIC3 that is most important to drive cell invasion. Indeed, we can recapitulate all of stromal CLIC3 pro-invasive capabilities simply by adding purified recombinant CLIC3 to the extracellular milieu at a concentration that is commensurate with the quantities of CLIC3 released by iCAFs (Supplementary Fig. 8c). CLIC proteins have been cast in a variety of guises including Cl^−^ channels and molecular scaffolds^28^, but our work corroborates the compelling evidence that their primary role in the cell is to function as oxidoreductases. A recent study has demonstrated that CLIC1,2 and CLIC4 have glutaredoxin-like activity, with the conserved cysteine at the N-terminal GST fold acting as the key catalytic residue^29^. Here we demonstrate that CLIC3 is also a GSH-dependent oxidoreductase and that cysteine 22 is the necessary active site. The cysteine 22 was necessary for all of TGM2-dependent extracellular pro-invasive functions of CLIC3. In combination with the fact that CLIC3 controls the GSH-dependent reduction of TGM2 at specific cysteines, and that CLIC3 influences the binding of TGM2 to its regulatory cofactors, our work supports the view that the pro-invasive capabilities of extracellular CLIC3 are imparted via its GSH-dependent oxidoreductase characteristics. Reactions catalysed by glutaredoxin-like enzymes depend on the redox context of the environment. In the strongly reducing environment of the cytosol, high GSH concentration (0.5–10 mM) can compromise protein activity by glutathionylation, and CLICs may de-glutathionylate these cysteine residues to restore protein activity^29^. However, outside the cell, where GSH concentrations are much lower (μM range), CLICs would not need to function as de-glutathionylating enzymes. Certain extracellular enzymes possess cysteine residues that control their activity, and reduction of oxidized cysteines in extracellular proteins is known to be performed by thioredoxins that use FADH as a source of reducing equivalents. TGM2 possesses cysteines 370 and 371 that must be reduced for the enzyme to be fully active, and thioredoxin has recently been shown to activate TGM2 by reducing these cysteines^37^. Like thioredoxins, GSH transferases catalyse reduction of cysteine using GSH as a source of reducing equivalents. We propose that CLIC3 acts in this way to activate extracellular TGM2. Intriguingly, cysteines 370 and 371 showed only a modest increase in reduction when TGM2 was in the presence of the enzymatically active CLIC3. Conversely, cysteine 505 was most reduced by CLIC3. Together with the fact that CLIC3 was able to alter TGM2 binding to its cofactors, and that cysteine 505 is conserved among species, our work opens to the possibility of alternative regulatory mechanisms of TGM2 activity.

We show that iCAFs silenced for CLIC3 or TGM2 generate an ECM with significantly reduced stiffness and that this can be almost completely restored simply by adding soluble purified recombinant CLIC3 (rCLIC3) to the culture medium, but not when silenced for TGM2. Moreover, CLIC3 ability to drive invasion depends on α_5_β_1_ integrin engagement. It is tempting to speculate that, consistent with the established role for α_5_β_1_ integrin in enhancing cell invasion by responding to microenvironmental stiffness^6^,^49^, CLIC3 acts via TGM2 γ-glutamyl crosslinking activity to drive ECM stiffness. Our polarized fluorescence experiments indicate that CLIC3 is capable of altering TGM2 association with its ligand GTP. Considering that α_5_β_1_ integrin activation drives fibronectin fibrillogenesis^50^ and that this may, in turn, increase ECM stiffness, an alternative intriguing hypothesis is that CLIC3 may influence the capacity of TGM2-GTP complex to bind to integrins. This may contribute to the α_5_β_1_ dependence of CLIC3-driven invasiveness in both tumour and endothelial cells (Fig. 8f).

Our study discovered an unprecedented molecular mechanism used by CAFs and cancer cells to generate a pro-invasive stroma, and opens up the possibility for the development of inhibitors of CLIC3 oxidoreductase activity to alter vessel growth and oppose tumour invasiveness. Moreover, we provide a comprehensive categorization of molecules responsible for generating a pro-angiogenic and pro-invasive stroma for the discovery of other pathways key to generate invasive cancers.

## Methods

### Cell culture

The iNF and iCAF cell lines (kindly provided by Professor Akira Orimo, Paterson Institute, Manchester) were cultured in Dulbecco's modified Eagle's medium (DMEM; Life Technologies) supplemented with 10% fetal bovine serum (FBS; Life Technologies). SILAC-labelled iNF and iCAF were cultured in SILAC DMEM (without arginine and lysine, Life Technologies) supplemented with 84 mg l^−1 12^C_6_^14^N_4_ L-arginine and 146 mg l^−1 12^C_6_^14^N_2_ L-lysine (that we refer to as ‘light', Sigma-Aldrich) or 84 mg l^−1 13^C_6_^15^N_4_ L-arginine and 175 mg l^−1 13^C_6_^15^N_2_ L-lysine (that we refer to as ‘heavy', Cambridge Isotope Laboratories), or 84 mg l^−1 13^C_6_^14^N_4_ L-arginine and 175 mg l^−1^ D_4_ L-lysine (that we refer to as ‘medium', Cambridge Isotope Laboratories), 92.1 mg l^−1^ L-proline (to reduce arginine to proline conversion, Sigma), 2% FBS and 8% 10 kDa dialyzed FBS (PAA). HUVECs were isolated and pooled from different umbilical cords (2–5) and cultured in EGM-2 (Lonza) for maximum 5 passages. MCF10DCIS.com breast cancer cells were kindly provided by Professor Philippe Chavrier and cultured in F12 (Gibco) 5% horse serum (Gibco). A2780 cells wild type or overexpressing Rab25 (kind gift from Dr Gordon Mills, MD Anderson Cancer Centre, Houston, TX, USA) were cultured in RPMI (Life Technologies) supplemented with 10% FBS. Mouse telomerase immortalized fibroblasts and MDA-MB-231 breast cancer cells (from ATCC) were cultured in DMEM supplemented with 10% FBS. Primary mammary CAFs were from the NHS-Glasgow biorepository (from LREC 01/63, R and D project 02PA002) or they were isolated from patient samples obtained through NHS Greater Glasgow and Clyde Biorepository. All participants gave specific consent to use their tissue samples for research. The pCAFs were cultured on collagen (35 μg ml^−1^ in PBS, rat tail Collagen I, BD Biosciences)-coated culture dish in DMEM 10% FBS. All cell lines are routinely tested for Mycoplasma at the facility available at the CRUK Beatson Institute.

### iCAF-iNF sample preparation for MS analysis

For the cell proteome, forward or reverse experiments (experiment 1, results are shown in the Figures and Supplementary Data 1): one 10 cm dish/cell type of 80–90% confluent cells was lysed in SDS buffer, 4% SDS, 100 mM dithiothreitol, 100 mM Tris HCl pH 7.6. Then, 100 μg of light and heavy lysates from iCAF and iNF were mixed together, boiled at 95 °C for 5 min sonicated using a metal tip (Soniprep 150, MSE) and centrifuged 16,000 *g* for 10 min. Proteins were then trypsin digested using the filter-aid sample preparation (FASP) method and 50 μg of peptides separated by strong anion exchange chromatography on StageTip as previously described^20^. Two additional biological replicates were performed, experiment 2 and experiment 3, where 50 μg of light and heavy lysates from iNF and iCAF or 50 μg of heavy and medium lysates from iNF and iCAF were mixed together, separated on 4–12% gradient NuPAGE Novex Bis-Tris gel (Life Technologies) and in-gel digested (20 gel slices)^51^. Results of experiments 2 and 3 are shown in Supplementary Data 1 (column ‘Ratio iCAF/iNF Cell Experiment 02 (log2)' and ‘Ratio iCAF/iNF Cell Experiment 03 (log2)').

For the cell-derived ECM, the ECM was prepared as previously described^21^,^52^ with minor modifications. Briefly, one 10 cm dish/cell type was cultured at confluence for 8 days in DMEM 10% FBS supplemented with 50 μg ml^−1^ ascorbic acid. Cells were removed by washing thoroughly with a buffer 20 mM NH_4_OH, 0.5% Triton X-100 in PBS and DNA digested with 10 μg ml^−1^ DNase I (Roche). The ECM was then lysed in SDS buffer, boiled at 95 °C for 5 min, sonicated using a metal tip (Soniprep 150, MSE) and centrifuged 16,000 *g* for 10 min. Equal volumes of SILAC heavy and light SDS soluble proteins from iNF and iCAF were mixed together and one-third of the sample separated on 4–12% gradient NuPAGE Novex Bis-Tris gel (Life Technologies) and in-gel digested (10 gel slices)^51^.

For the conditioned medium, one 10 cm dish of 90% confluent cells was washed thoroughly with PBS and incubated for 24 h in 7 ml DMEM without serum. Then, 5 ml of each SILAC heavy and light conditioned medium from iNF and iCAF were mixed together. After three-step centrifugation at 4 °C of the mixed sample, 10 min at 300 *g*, 10 min at 2,000 *g* and 30 min at 10,000 *g*, the cleared supernatant was acidified to pH 5.0 (optimal pH for efficient recovery of proteins from the conditioned medium, Supplementary Fig. 1a) with 10% trifluoracetic acid and incubated with 100 μl of Strataclean-resin (Agilent Technologies) for 60 min rotating on a wheel at room temperature (RT). Proteins bound to the beads were eluted with 100 μl of loading buffer (Bio-Rad), boiled 5 min at 95 °C and one-third of the sample separated on 4–12% gradient NuPAGE Novex Bis-Tris gel (Life Technologies) and in-gel digested (7 gel slices)^51^.

### MS analysis of iCAF-iNF proteomes

Digested peptides were desalted using StageTip^53^. After removal of acetonitrile (ACN) using speed vacuum, peptides were resuspended in 1% trifluoracetic acid, 0.2% acetic acid buffer and injected on an EASY-nLC system coupled on line to a LTQ-Orbitrap Velos via a nanoelectrospray ion source. Peptides were separated using a 20 cm fused silica emitter (New Objective) packed in house with reversed-phase Reprosil Pur Basic 1.9 μm (Dr Maisch GmbH) and eluted with a flow of 200 nl min^−1^ from 5 to 30% of buffer containing 80% ACN in 0.5% acetic acid, in a 90 min linear gradient (190 min gradient for the cell proteome). The full-scan MS spectra were acquired in the Orbitrap at a resolution of 30,000 at *m/z* 400. The top 10 most intense ions were sequentially isolated for fragmentation using high-energy collision dissociation, and recorded in the Orbitrap at resolution of 7,500. Data were acquired with Xcalibur software (Thermo Fisher Scientific). An active background ion reduction device (ABIRD, SmartSource Solutions, LLC) was used to remove background ions. The MS files were processed with the MaxQuant software^22^ version 1.3.8.2 (MS files from cells, ECM and CM proteome were analysed together) and searched with the Andromeda search engine^54^ against the human UniProt database^55^ (release-2012 01, 88,847 entries). To search the parent mass and fragment ions we required an initial mass deviation of 4.5 p.p.m. and 20 p.p.m., respectively. The minimum peptide length was set to seven amino acids and a maximum of two missed cleavages and strict specificity for trypsin cleavage were required. Carbamidomethylation (cysteine) was set as fixed modification, whereas oxidation (methionine) and *N*-acetylation were set as variable modifications. The false discovery rates at the protein and peptide level were set to 1%. The scores were calculated as described previously^54^. The requantification and match between runs features were enabled and the relative quantification of the peptides against their SILAC-labelled counterparts was performed by MaxQuant. For protein group quantification unique and razor peptides (=most likely belonging to the protein group) were used and we required proteins to be quantified with at least two ratio counts.

### MS data analysis of iCAF-iNF proteomes

The common reverse and contaminant hits (as defined in MaxQuant) were removed from the MaxQuant output and the normalized SILAC ratios were further normalized to the median.

Only protein groups identified with at least one uniquely assigned peptide were used for the analysis. Protein groups were considered reproducibly quantified in the cells, ECM and CM fractions if identified and quantified in forward and reverse experiment. For forward and reverse experiment, protein groups with significant SILAC ratio were determined according to the Significance B as described in ref. 54, using 5% as false discovery rate. Protein groups with different abundance between iNF and iCAF are those that passed the significance B test in the forward or reverse experiment and hits with opposite regulation in the two experiments were excluded.

Categorical annotation of protein groups was performed with the Perseus module of MaxQuant^56^ that uses annotations according to Uniprot.

Functional and interaction network was generated by querying STRING version 9.1 (ref. 26) using the UniProt ID of the proteins significantly different between iNF and iCAF (iCAF signature of 325 proteins) using the parameters Active prediction method: Experiments, Databases, Neighbourhood and Score: 0.400. The identified protein–protein connections were uploaded into Cytoscape^57^.

### Ovarian and breast cancer TMAs

CLIC3 staining was evaluated in breast cancer patient samples from TMAs that have been previously described in refs 30, 31. The ovarian TMA 2 has been previously described in ref. 58. Ovarian cancer TMA 1 was produced at the Molecular Histopathology Services Laboratory Medicine Unit, Southern General Hospital in conjunction with the NHSGGC Biorepository, Southern General Hospital. The study was approved by the West of Scotland Research Ethics Service REC 4. REC reference: 10/S0704/60. The tissue microarray was created with triplicate cores from formalin-fixed, paraffin-embedded tumour material collected at either upfront primary debulking surgery or delayed primary surgery. All participants gave specific consent to use of archival material for future translational research. A total of 201 cores from 67 patients were arrayed on the TMA, of those 24 were HGS, 22 clear cell and 21 endometrioid ovarian cancers.

CLIC3 immunohistochemistry and use of the CLIC3 histoscore calculation in cancer cells have been described previously in ref. 31. The CLIC3 histoscore in the stroma was blindly performed by two investigators: nontumour cell tissue was considered as stroma and the score (no, low, medium and high) represents the intensity of CLIC3 staining in the majority of the positively stained stroma. The percentage of positive stroma was not included in the histoscore.

### Microarrays analysis

Microarrays data of cultured oral NFs and CAFs were from GEOD-35356 (12 healthy people undergoing plastic surgery and 12 OSCC patients undergoing surgical resection) or GEOD-38517 (fibroblasts derived from dysplastic oral mucosa and oral squamous cell carcinoma compared with fibroblasts derived from normal oral mucosa); of laser capture microdissected (LCM) ovarian cancer normal and tumour stroma were from GSE40595 (31 LCM cancer-associated stroma samples, 32 epithelial tumour samples from high-grade serous ovarian cancer patients, 8 microdissected normal ovarian stroma samples and 6 human ovarian surface epithelium (cell samples); of LCM colorectal cancer normal and tumour stroma were from GSE35602 (tissue samples from 13 colorectal cancer tissues and 4 normal tissues were microdissected using Laser Microdissection System (Leica Microsystems), and RNA samples specific for stroma or epithelium were separately collected); of cultured colorectal NF and CAF were from GEOD-46824 (we obtained fibroblast cultures from fresh surgical specimen ressected from patients with primary colorectal carcinoma: normal colonic fibroblasts (9) from the normal colonic mucosa at least 5–10 cm from the surgical margin, carcinoma-associated fibroblasts from the primary tumour (CAF-PT=14) and carcinoma-associated fibroblasts (CAF-LM=11) from fresh surgical specimens of liver metastases); of cultured prostate NFs and CAFs were from GSE34312 (Stromal cells cultured from normal peripheral zone tissues (F-PZ-64, F-PZ-79, F-PZ-82, F-PZ-102, F-PZ-105) and from tumours (F-CA-31, F-CA-39, F-CA-52, F-CA-67, F-CA-93) were established and grown as previously described in Peehl et al. (2000)^59^. Total RNAs were extracted from semiconfluent cells (passages 4–5) 1 day after feeding fresh medium).

### Atomic force microscopy

Force indentation measurements were carried out using AFM colloidal probes that were prepared by attaching 4.8 μm spherical silica beads to tipless Nanoworld TL1 cantilevers (spring constant =0.02 N m^−1^)^60^. Calibration measurements were performed before every experiment to deduce the spring constant for each cantilever. All measurements were undertaken in fluid at a speed of 5 μm s^−1^ with a loading force of 3 nN. The Young's modulus was extracted from the force indentation curves using the Hertz model^60^. Considering the Hertz model is generally valid for small indentations, which are in the region of <15% of the total sample thickness. All curves were analysed up until a 300 nm depth limit, even if the total curve depth extended deeper.

### 3D endothelial cell fibrin gel

The 3D fibrin gel HUVEC-fibroblast co-culture has been performed in 24-well/plate as previously described^61^, with minor modifications, such as that HUVECs had always been cultured in EGM2 and fibroblasts in DMEM 10% FBS. For the reagents, collagen-coated beads (Cytodex 3; Cat. No. 17-0485-01) were from Amersham, and Aprotinin, Fibrinogen type 1 and Thrombin were from Sigma Aldrich. Medium was changed (including treatments were indicated) every second day. Bright-field images were acquired with an Axiovert 25 microscope (Zeiss) equipped with a Retiga EXi Fast 1394 camera (Imaging). Lumens were discerned by visual inspection of bright-field images.

### Tumour cell pseudopod length

A2780 ovarian carcinoma cells or MDA-MB-231 cells were plated onto the fibroblast-derived ECM in 6-well plate at a density of 1 × 10^5^ cells per well. After 2 h, indicated stimuli were added in the medium and 3 h later the time lapse started. Pictures of the cells were taken every 5 min over a 22 h period with a 10 × objective on a Nikon Eclipse Ti microscope equipped with a CoolSNAP HQ CCD camera (Photometrics). Cells were maintained at 37 °C and 5% CO_2_ for the duration of the experiment (environmental control chamber, Okolab). The pseudopod length analysis was carried out with ImageJ. The pseudopod length was measured from the nucleus to the frontal tip of the cell. For each well, six fields were recorded and for each of them the length of the pseudopod of 30 cells measured (=180 cells measured for each experimental condition in each replicate experiment). For the experiment where iNF- and iCAF-generated CM was used as stimulus (Fig. 1), only MDA-MB-231 cells were used because these cells grow in the same medium (DMEM) as the fibroblasts.

### Inverted tumour cell invasion assay

Matrigel invasion assay was performed as described previously^62^. Briefly, A2780 cells were seeded on the base of transwell (8 μm, Corning) coated with a thick Matrigel solution composed by pure growth factor reduced geltrex (LDEV-Free Reduced Growth Factor Basement Membrane Matrix, Cat. No. A1413202) diluted 1:1 in cold PBS and supplemented with 25 μg ml^−1^ soluble fibronectin and stimuli. Tumour cells were allowed to migrate towards a gradient of 10% FCS and 25 ng ml^−1^ epidermal growth factor for 72 h. Invasion was measured by labelling cells with 4 μM acteoxymethyl ester calcein followed by confocal microscopy serial sections analysis using an Olympus FV1000 equipped with an argon laser (SLC), a photon multiplier tube (Hamamatsu Photonics) and a 20 × objective. For each individual experiment three transwells per condition were analysed and three different optical sections were taken from three areas of each transwell.

### 3D MCF10DCIS.com cancer cell invasion

To investigate the role of CLIC3 in tumour cell invasion, we use ER^−^ MCF10DCIS.com mammary cells that are derived from the ‘normal' MCF10A cell line and form well-defined comedo-like DCIS when injected as xenografts. However, these lesions spontaneously progress to invasive carcinoma at a predictable rate^42^,^63^. Elements of this progression may be recapitulated in 3D culture^41^. Indeed, when cultured in matrigel for up to 5 days, MCF10DCIS.com cells formed noninvasive comedo structures (referred to as mammosphere) bounded by a basement membrane as determined by immunofluorescence staining for laminin 5 and β4 integrin^30^. Following 6 days of culture, these comedo-like structures began to lose their sphericity as the basement membrane lost integrity and the mammospheres became spontaneously more invasive. Mammosphere were generated as described in refs 30, 64. Briefly, 5 × 10^3^ MCF10DCIS.com cells per well of an eight-well chamber slide were plated on a 40 μl layer of Matrigel (5 μl for immunofluorescence analysis) as previously described. Multiple phase-contrast images at × 10 magnification were captured from duplicate wells after 6 days of culture and circularity was determined using ImageJ.

### Aortic ring assay

The aortic ring angiogenesis assay has been performed as previously described^65^,^66^ with minor modifications. Briefly, thoracic aortas were isolated from 8-week-old C57Bl/6J mice (Charles River Research Models & Services) that were euthanized by cervical dislocation. Under a dissection microscope, surrounding fat, tissue and branching vessels were removed and aortas were cut in ∼0.5 mm thick rings and put in culture dish, covered with Opti-MEM (Life Technologies) and cultured for 48 h at 37 °C in 5% CO_2_. Each ring was then separately embedded into a 20 μl drop of 1.6 mg ml^−1^ collagen rat tail solution (Cat. No. 11179179001, Roche) in EBM-2 (Lonza), pH 7, deposited onto a glass bottom Microwell plate (MatTek). After 30 min of incubation at 37 °C to allow the collagen to solidify, 2 ml of EBM2-containing heparin, 10% FBS, containing 30 ng ml^−1^ murine VEGF_165_ (PreproTech) and either vehicle or 25 ng ml^−1^ purified human recombinant rCLIC3 was gently added to the plate. Medium was replaced every second day and images captured 6 days after embedding. Mice were housed in individual ventilated cages in a barrier animal facility proactive in environmental enrichment. All animal work was done in accordance with ethical approval from University of Glasgow under the revised Animal (Scientific Procedures) Act 1986 and the EU Directive 2010/63/EU authorized through Home Office Approval (Project licence number 60/4181).

### Matrigel plug assay

Growth factor reduced phenol red free matrigel (Corning) in liquid form at 4 °C was mixed with vehicle (50 mM Tris HCl pH 7.4) or FGF2 15 U ml^−1^ heparin or purified human recombinant CLIC3 (wild type or C22A mutant, 500 ng) alone or in combination. For TGM2 inhibition, Z-DON (20 nM) was additionally added to the matrigel solution. Matrigel (0.5 ml) was injected into the abdominal subcutaneous tissue of female BALB/c-nude mice (8 weeks old, 6 mice per group, 3 or 4 groups) along the peritoneal midline. On day 10, plugs were harvested, weighted and divided for haemoglobin measurement and immunohistochemical analysis (fixed overnight in 2% paraformaldehyde (PFA)). Vascular identity of infiltrating cells was established by Pecam1 (BD Bioscience) immunostaining. Matrigel plug haemoglobin content was measured using the Drabkin method (Drabkin reagent kit, Sigma) according to the manufacturer's recommended protocol. Mice were randomly assigned to a treatment group and the investigator was blinded when assessing the outcome. All mouse procedures were approved by the Institutional Animal Care and Research Advisory Committee of the K.U. Leuven. Animals were excluded from the analysis if they were considered outliers based on Prism analysis.

### Xenograft

The 8-week-old female BALB/c-nude mice (Charles River) were subcutaneously injected in the flank with 5 × 10^5^ MCF10DCIS.com cells (at P11) that had been resuspended in 400 μl of which 200 μl was growth factor reduced phenol red free matrigel (BD Bioscience) and 200 μl was vehicle (PBS) alone or in combination with human purified recombinant CLIC3 (500 ng), with or without Z-DON (20 nM). Mice were humanely euthanized after 15 days from inoculation and tumours excised and used for immunohistochemical analysis. The circularity of the tumours was determined using ImageJ. Only tumours with a peripheral laminin 5 staining were quantified. Mice were randomly assigned to a treatment group and the investigators were blinded when assessing the outcome. Mice were housed in individual ventilated cages in a barrier animal facility proactive in environmental enrichment. All animal work was done in accordance with ethical approval from University of Glasgow under the revised Animal (Scientific Procedures) Act 1986 and the EU Directive 2010/63/EU authorized through Home Office Approval (Project licence number 60/4181). Animals were excluded from the analysis if they were considered outliers based on Prism analysis.

### Recombinant proteins generation and purification

For the GST-CLIC3 and GST-CLIC3^C22A^ constructs, the coding region of CLIC3 with flanking restriction sites (see table below for primers and restriction sites) was amplified and subcloned into the expression vector pGEX-6P-1. Mutation of the cysteine at position 22 with an alanine was performed using QuickChange site-directed mutagenesis (see Table 2 for primers) according to the manufacturer's instructions (Stratagene).

*E. coli* BL21 (DE3) pLysS cells (Invitrogen) transformed with pGEX-6P-1-CLIC3 vector were grown at 37 °C until the cell density reached an OD_600_ of 0.6, at which point GST-CLIC3/CLIC3^C22A^ expression was induced with 0.25 mM isopropyl β-D-thiogalactosidase (IPTG) at 30 °C for 2 h. The bacteria were harvested by centrifugation at 3,300 *g* at 4 °C for 1 h. Cell pellets were resuspended in 100 ml of lysis buffer containing 0.1% Triton X-100, 2 mM Benzamidine, 3 μM Pepstatin, 3 μM Antipain, 4 μM Leupeptin and 0.3 μM Aprotinin in PBS pH 7.4. The identity of rCLIC3 and rCLIC3^C22A^ was verified by MS-based analysis. Samples were in-gel digested with trypsin^51^ and analysed on a LTQ-Orbitrap Velos using operated in the Collision Energy Dissociation (CID) mode to fragment the peptides. Data were analysed with the MaxQuant computational platform^22^.

For GST-TGM2 construct, *E. coli* BL21 (DE3) pLysS cells (Invitrogen) transformed with pGEX-6P-1-TGM2 vector (kindly provided by Professor Jeffrey Keillor) were grown at 25 °C until the cell density reached an OD600 reading of 0.6, at which point the temperature was reduced to 18 °C before overnight induction with 1 μM IPTG. The bacteria were harvested by centrifugation at 3,300 *g* at 4 °C for 1 h. Cell pellets were resuspended in 100 ml of lysis buffer containing 20 mM Tris HCl pH 8, 150 mM NaCl, 1 mM EDTA, 1 mM TCEP pH8, 15% glycerol and 2 tablets of Complete ultra protease inhibitors without EDTA (Roche).

For the purification of the recombinant proteins, the bacterial suspension was incubated with DNAase (30 μg) on ice for 15 min, homogenized in a Microfluid instrument (Model M-110 P) and centrifuged at 33,000 *g* at 4 °C for 1 h. The GST-tagged recombinant proteins were purified using GSTrap HP chromatography (GE Healthcare Life Sciences). The GST was cleaved from the purified proteins *in situ* by proteolysis with PreScission protease (Life Technologies) according to the manufacturer's instructions, and the recombinant GST-free proteins eluted, concentrated (Amicon ultra 15, 10 kDa, Millipore) and stored at −80 °C until use.

### Quantitative MS analysis of reduced cysteines

The buffer that TGM2 was stored in was exchanged with PBS before experiment using a desalting column (Zeba Spin Desalting Columns, Thermo Scientific); then, TGM2 was oxidized with 0.5 μM diamide and the diamide removed using a desalting column. Oxidized rTGM2 was incubated with rCLIC3 or rCLIC3^C22A^ in the presence of 10 μM GTP, with or without 1 mM GSH for 20 min at 37 °C. To measure the levels of reduced cysteines in rTGM2 between experimental conditions (details of the protocol: Lilla and Zanivan, manuscript in preparation), after the reaction, samples were treated either with light iodoacetamide (IAA) or with stable isotope containing IAA (Sigma). Samples were then mixed together, precipitated with trichloroacetic acid and digested with trypsin. For each experiment, two independent replicates were performed, switching the labelling conditions. Samples were run on a LTQ-Orbitrap Velos or on a Q-Exactive HF coupled on line to an EASY-nLC 1200 system (Thermo Fisher Scientific). Peptides were eluted with a flow of 200 nl min^−1^ (EASY-nLC) or 300 nl min^−1^ (EASY-nLC 1200) from 5 to 27% (EASY-nLC) and from 2 to 20% (EASY-nLC 1200) of buffer containing 80% ACN in 0.1% formic acid in a 42 min linear gradient. The full-scan MS spectra were acquired in the Orbitrap at a resolution of 60,000 at *m/z* 200. The top 10 most intense ions were sequentially isolated for fragmentation using high-energy collision dissociation, and recorded in the Orbitrap at resolution of 15,000 at *m/z* 200. The MS. RAW data were acquired with Xcalibur software and processed with the MaxQuant software^22^ version 1.5.3.30 and searched with the Andromeda search engine^54^ against an in-house database containing common contaminants and human TGM2, CLIC3 and CLIC3^C22A^ sequences (1,961 entries). Msms.txt file from MaxQuant was imported into Skyline to extract the XICs (60 K resolution at 400 *m/z*) of the cysteine-containing peptides of TGM2 that carried 2+ and 3+ charges and that were modified with IAA light and heavy. Extracted XIC were used for quantification.

### Polarized fluorescence

To measure the functional/physical interaction between TGM2 and CLIC3, fluorescence-based polarization measurements were used. This approach exploits the capability of TGM2 to bind to GDP/GTP and uses the nucleotide analogue MANT-GMPPNP (Life Technologies), where the modified ribose moiety has been shown to minimally interfere with the binding between protein and nucleotide. For this assay, recombinant TGM2 and CLIC3 expressed as GST-tagged protein in *E. coli* and purified using affinity chromatography followed by removal of the GST tag were used (see Methods above). The evaluation of the interaction between CLIC3 and TGM2 operates on the principle that the binding of another moiety to the Mant-GMPPNP.TGM2 complex will be reflected in an altered fluorescence polarization signal. Fluorescence polarization measurements were performed at RT in 20 mM Tris-HCl pH 8, 150 mM NaCl, 1 mM EDTA, 1 mM TCEP and 15% glycerol buffer, containing 2 μM MANT-GMPPNP, 1 mM GSH (Fisher Scientific), 1 mM GSSG (Sigma-Aldrich), 10 mM CaCl_2_ (Sigma-Aldrich), 2 μM unlabelled GTP and purified recombinant human proteins TGM2, CLIC3 and CLIC3^C22A^ at the indicated concentrations alone or in combination. Data were recorded with a Photon Technology International fluorimeter (PTI, equipped with LPS-220B, Bryteleox, SC-500, MD-5020 and photomultiplier detection system 814 modules and Felix 32 analysis V 1.2 software), with excitation and emission wavelengths at 355 and 440 nm, respectively, for MANT-GMPPNP.

### HEDS enzyme assay

The reduced monomeric CLIC1 wild type, CLIC1^C24A^ mutant, CLIC3 wild type or CLIC3^C22A^ mutant (10 μM final concentration) was added to 5 mM potassium phosphate buffer (pH 7) containing 1 mM EDTA, 250 μM NADPH, 50 nM GR and 1 mM HEDS. The mixture was incubated for 5 min at 37 °C, with the reaction initiated by addition of 1 mM GSH. Consumption of NADPH was monitored at A340 nm.

### Western blot

Proteins were separated on 4–12% gradient NuPAGE Novex Bis-Tris gel (Life Technologies), transferred to PVDF membrane (Millipore), blocked in 1 × TBS-Tween with 5% non-fat dry milk and incubated with corresponding primary antibody and secondary antibodies. The following antibodies were used: rabbit anti-TGM2, (1:1,000; Cat. No. HPA021019, Sigma-Aldrich, Prestige Antibodies Powered by Atlas Antibodies), Rabbit anti-CLIC3 (1:3,000; produced in house^31^), Rabbit anti-β-tubulin (1:1,000; Cat. No. sc-9104, Santa Cruz), Mouse anti-Vinculin (1:1,000; Cat. No. V9131, Sigma-Aldrich) and Mouse anti-αSMA (1:1,000; Cat. No. A5228, Sigma). As secondary antibodies, horseradish peroxidase-conjugated (1:10,000; Cat. No. HAF008 and HAF007, H&D systems,), IRDye 680RD (1:10,000; Cat. No. 926-68072, LI-COR) and IRDye 800CW (1:10,000; Cat. No. 926-32213, LI-COR) were used. Images were captured with a Bio-Rad GS-800 Calibrated densitometer (Quantity-One software version 4.6.3) or a LI-COR Odyssey CLx scanner (Image Studio software, version 5.0.21) for chemluminiscent or fluorescence western blots, respectively. Representative images from reproducible, independent experiments are shown. Uncropped scans of the western blots are reported in Supplementary Information (Supplementary Figs 9 and 10).

### Small interfering RNA

The day of transfection, iCAF were 70–80% confluent. The Amaxa electroporation kit R (Lonza) for transfection of the siRNA was used according to the manufacturer's protocol. Briefly, 3 × 10^6^ iCAFs were transfected with 1 nM nontargeting siRNA or specific siRNA for CLIC3 or TGM2 (Dharmacon-Thermo). SiCLIC3=5′-CGGACGUGCUGAAGGACUU-3′ (refs 30, 31); TGM2=Smart Pool. For primary fibroblasts experiment, cells were transfected with oligofectamine (Invitrogen, Life Technologies) according to the manufacturer's instructions. Experiments were performed 24 h after the transfection.

### Immunofluorescence

For the 3D MCF10DCIS.com cancer cell invasion, the staining was performed as previously described^30^. Briefly, cells were fixed with 2% PFA for 20 min at RT and permeabilized with 0.5% Triton for 10 min at RT and blocked with 1% bovine serum albumin. Staining was performed using the following antibodies: anti-laminin-5 (1:200; Cat. No. mab19562, Merck Millipore)^30^; anti-β4 integrin (1:200; Cat. No. BD555722, BD Biosciences)^68^; and Alexa Fluor 488 or 555-conjugated secondary antibodies (1:500; Molecular Probes Life Technologies).

### Immunostainings

Immunohistochemistry staining was performed on 4 μm thick sections of formalin-fixed, paraffin-embedded tissue of TMA and patient tissue samples following standard protocols. The antigen retrieval was performed with Sodium Citrate at pH 6.0. Sections were stained using Rabbit anti-CLIC3 (1:750; produced in-house^31^), rabbit anti-TGM2 (1:20; Cat. No. HPA021019, Sigma, Prestige Antibodies Powered by Atlas Antibodies), mouse anti-αSMA (1:25,000; Cat. No. A5228, Sigma)^69^ and mouse anti-laminin 5 (1:250; Cat. No. mab19562, Merck Millipore)^70^.

### Statistical analysis

Statistical analysis was carried out using GraphPad Prism software (GraphPad Software, Inc.). When comparing two samples, for 3D fibrin gel assay, endothelial motility, tumour cell invasion, AFM, CLIC3 score in TMA and microarrays, *P*-value was calculated according to Mann–Whitney test. For the pseudopod elongation, Kruskal–Wallis test and Dunn's multiple comparison test were used. For multiple samples comparison, *ex vivo* and *in vivo* experiments, and cell proliferation, *P*-value was calculated according to the two-tailed Student's *t*-test. Unless indicated otherwise, bars represent mean±s.e.m. and each experiment was performed a minimum of three times (biological replicates) and figures show one representative replicate.

### Data availability

The raw MS files and search/identification files obtained with MaxQuant have been deposited in the ProteomeXchange Consortium (http://proteomecentral.proteomexchange.org/cgi/GetDataset) via the PRIDE partner repository^71^ under accession code PXD002444. All the other data generated or analysed during this study are included in this published article (and its Supplementary Information files) or are available from the authors (on reasonable request).

## Additional information

**How to cite this article:** Hernandez-Fernaud, J. R. *et al*. Secreted CLIC3 drives cancer progression through its glutathione-dependent oxidoreductase activity. *Nat. Commun.* **8,** 14206 doi: 10.1038/ncomms14206 (2017).

**Publisher's note:** Springer Nature remains neutral with regard to jurisdictional claims in published maps and institutional affiliations.

## Supplementary Material

Supplementary Information Supplementary Figures, Supplementary Tables and Supplementary References

Supplementary Data 1 Proteins identified and quantified in the cell, conditioned medium (CM) and extracellular matrix (ECM) proteomes of SILAC-labelled iNF and iCAF. NaN = Not a number. It refers to proteins which have not been quantified

## Figures and Tables

**Table 1:** Overall survival of the HGS patients in TMA 1 and 2.

	HGS TMA 1		HGS TMA 2	
	AVG OS	SD OS	AVG OS	SD OS
*Stroma CLIC3 histoscore*				
Low (0)	n.a.	n.a.	9.8	7.3
Medium (5)	24.7	11.2	8.1	3.4
High (6)	17.4	13.6	4.0	2.2
				
*Tumour cells CLIC3 histoscore*				
Low (1)	41.5	n.a.	8.9	6.2
Medium (4)	20.5	7.0	5.8	3.9
High (6)	17.4	12.4	3.1	1.4

OS, overall survival, expressed in months.

**Table 2:** Primers for QuickChange site-directed mutagenesis.

**Description**	**Sequence**
CLIC3 C22/A22 Fw	5′-GAGAGCGTGGGTCAC**GCC**CCCTCCTGCCAGCGGCTCTTCATG-3′
CLIC3 C22/A22 Rev	5′-CATGAAGAGCCGCTGGCAGGAGGG**GGC**GTGACCCACGCTCTC-3′
CLIC3 *Xho*I Fw	5′-GTACTCGAGCTATGGCGGAGACCAAGCTC-3′
CLIC3 *Bam*HI Rev	5′-ATTGGATCCCTAGCGGGGGTGCAC-3′
