# Supplementary material for: Secreted CLIC3 drives cancer progression through its glutathione-dependent oxidoreductase activity
Source: Nat Commun. 2017 Feb 15;8:14206. doi: 10.1038/ncomms14206 (PMC5316871; doi:10.1038/ncomms14206)
Supplement: Supplementary Information — Supplementary Figures, Supplementary Tables and Supplementary References [file ncomms14206-s1.pdf]

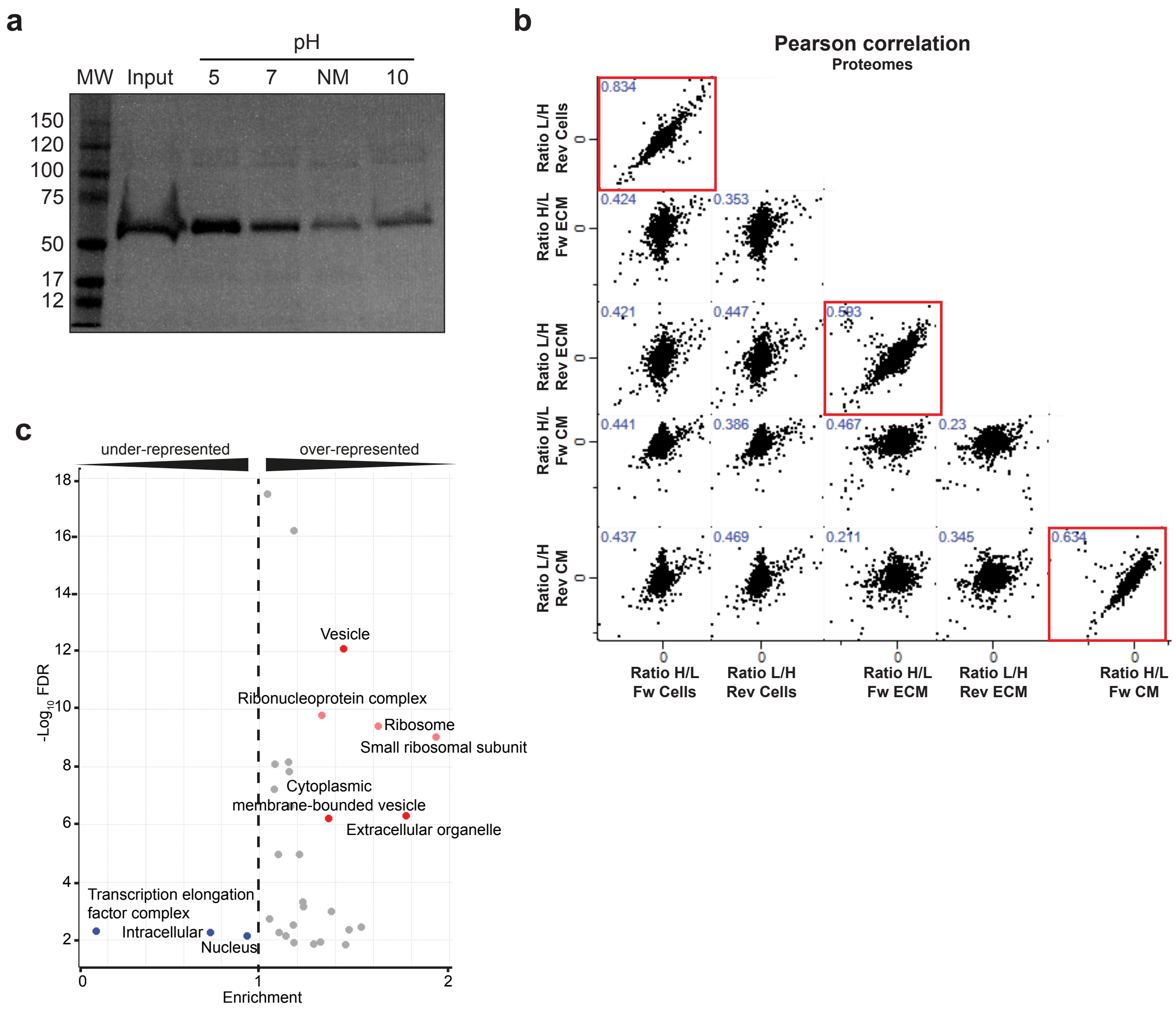

Supplementary Figure 1. Proteomic analysis of iNF and iCAF. (a) Comassie gel staining (according to manufacturer's instructions, Instant blue, Expedeon) showing the amount of BSA recovered with Strataclean-beads by incubating BSA-containing EBM2 medium (input) acidified with trifluoroacetic acid (TFA) at the indicated pH or non-modified (NM). (b) Pearson correlation coefficient analysis of the SILAC ratio iCAF/iNF (log_2) which shows high correlation between forward (Fw) and reverse (Rev) SILAC replicates (highlighted in the red squares). (c) Gene ontology cellular compartment (GOCC slim) enrichment analysis based on Fisher exact test (2% Benjamini-Hochberg FDR) performed with Perseus for the subset of proteins identified in all three fractions, Cell, ECM and CM proteome. The total proteome was used as background.

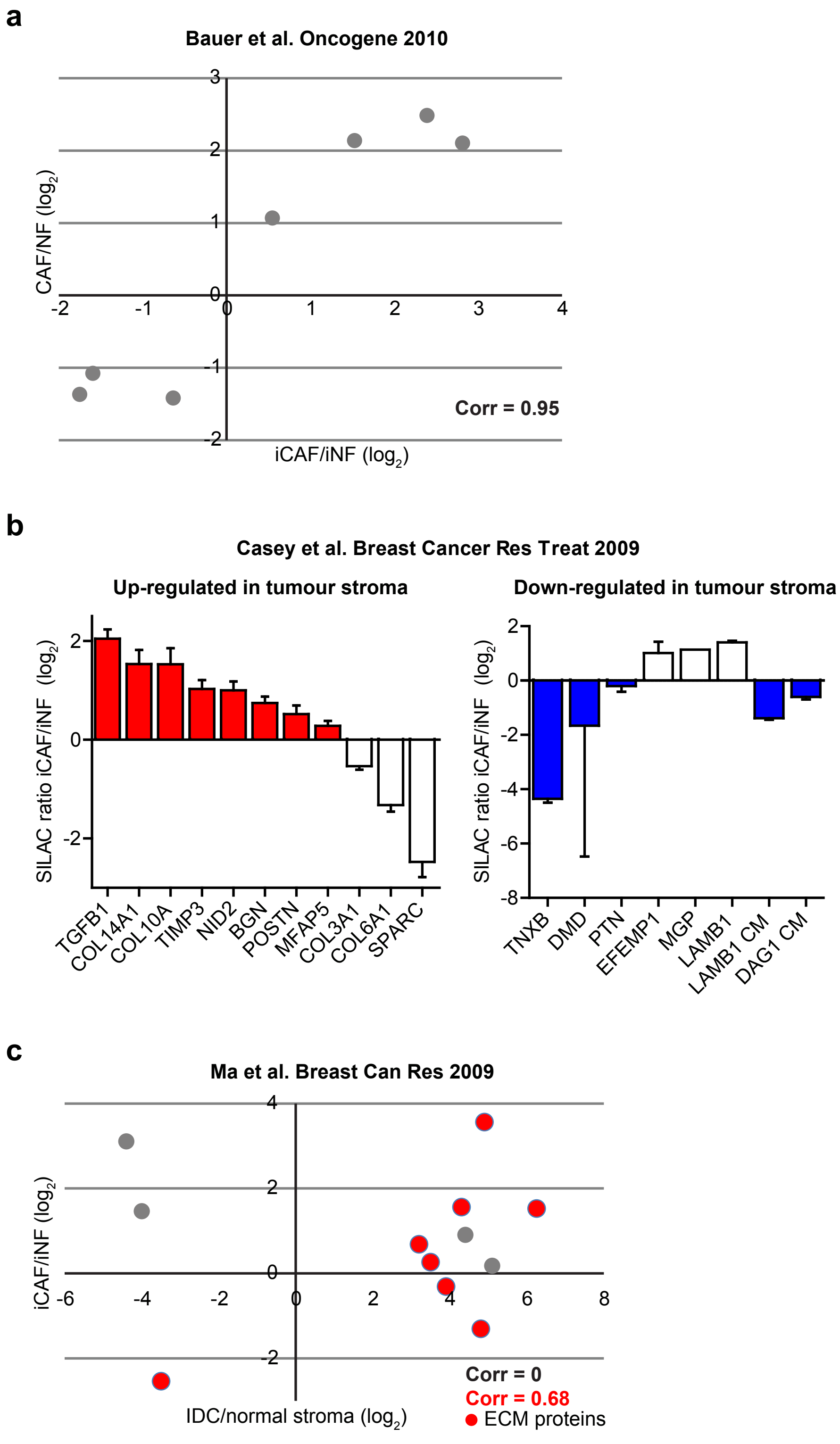

Supplementary Figure 2. Comparison between proteomics and gene expression data of breast normal and cancer stroma. (a) Comparison with the signature reported in (1), where patient-matched normal fibroblast (NF) and CAF were isolated from six breast cancer patients, cultured, and gene expression analysed. Corr = Pearson correlation coefficient. The protein levels/gene expression ratios calculated in the two studies are reported in Table 1. (b) Comparison with the signature reported in (2), where the gene expression of laser-captured micro-dissected stroma from normal breast tissue (5 patients) and invasive breast cancer (23 patients) was analysed. In the two plots the SILAC ratio iCAF/iNF of the proteins reported in the signature are shown. Red = proteins/genes upregulated in both studies; blue = proteins/genes downregu-lated in both studies. Bars = mean +/- SEM. (c) Comparison with the signature reported in (3) where the gene expression of patient-matched laser-captured micro-dissected normal stroma and stroma associated to invasive ductal carcinoma (IDC) of 14 patients was analysed. Measured averaged ratio values calculated in the two studies are reported in Table 2.

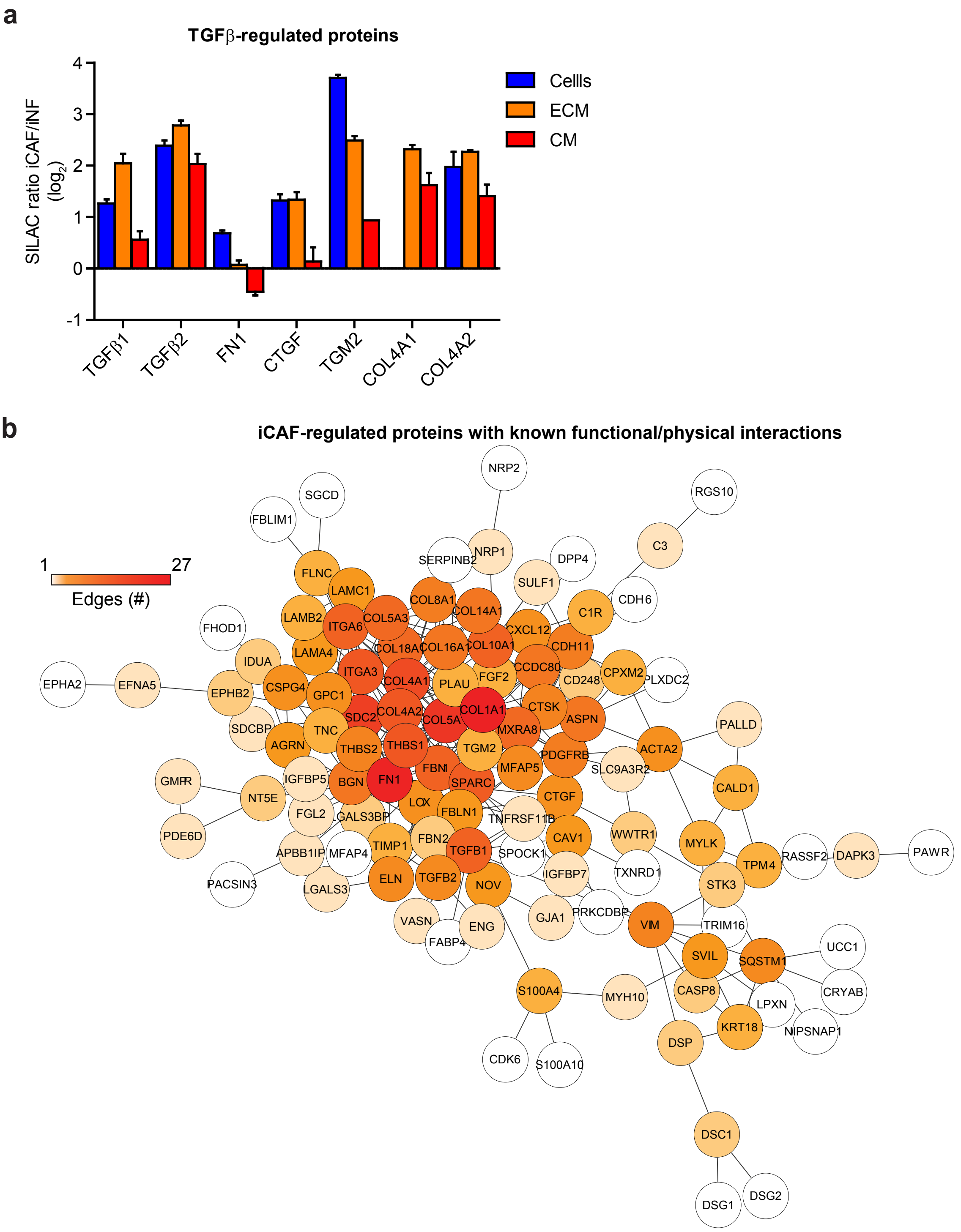

Supplementary Figure 3. Fibroblasts activation increases TGFβ signalling and levels of secreted proteins, including CLIC3. (a) SILAC ratio iCAF/iNF measured for TGFβ-regulated proteins quantified in the cell, ECM and CM proteomes. N = 2 SILAC experiments, forward and reverse. Bars = mean +/- SEM. (b) Most highly connected network of proteins identified by STRING and visualized with Cytoscape (4). The colour intensity of each node indicates the number of nodes it interacts with (= number of edges).

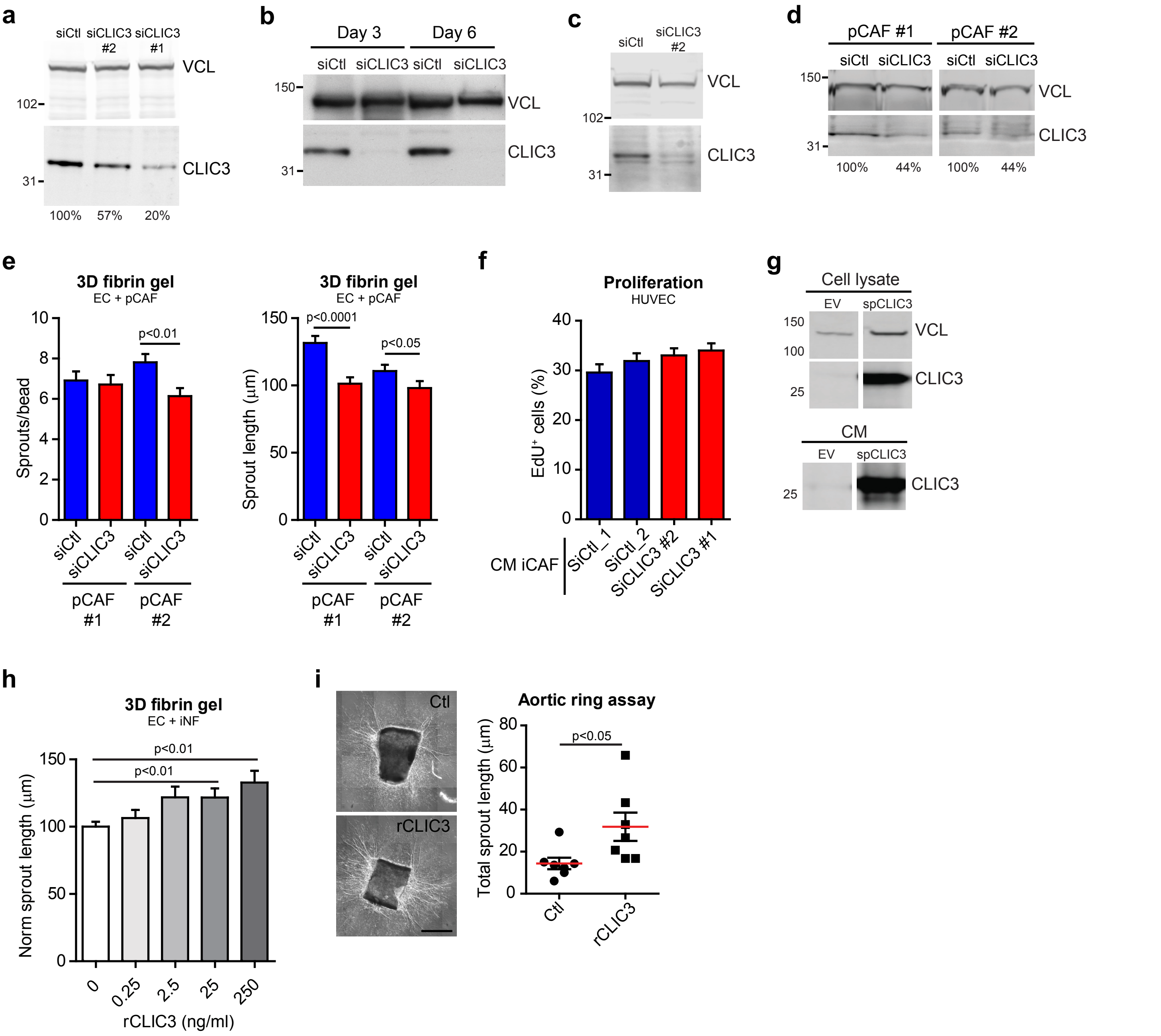

Supplementary Figure 4. CLIC3 is required for mammary CAF functions. (a) Western blot analysis for CLIC3 showing the efficient silencing upon transfection of iCAF with siRNAs specific for CLIC3. CLIC3 quantification (shown as % of measured CLIC3 compared to siCtl) based on LICOR software. These iCAF were used for testing cell proliferation in Fig. 4a. siCtl = non-targeting siRNA. VCL = Vinculin, used as loading control. (b,c) Western blot analysis for CLIC3 showing the efficient silencing after 3 and 6 days (b, siCLIC3 #1) or 6 days (c, siCLIC3 #2) from transfection with siRNA specific for CLIC3 in iCAF used for the 3D fibrin gel assay shown in Fig. 4b. (d) Western blot analysis for CLIC3 showing the efficient silencing after 4 days from transfection with siRNA specific for CLIC3 in cultured primary mammary CAF isolated from patient samples and used for the 3D fibrin gel co-culture assay in panel (e). CLIC3 quantification (shown as % of CLIC3 measured compared to siCtl) based on LICOR software. (e) Sprouting quantification, sprout number and length, of HUVEC embedded into 3D fibrin gel and co-cultured with human primary mammary CAFs (pCAFs), which were isolated from two patients (#1 and #2), transfected with Oligofectamine (Invitrogen), according to manufacturer's instruction, with siRNA for CLIC3 or non-targeting siRNA. Bars = mean +/- SEM. $n_{CtlpCAF\#1} = 47$, $n_{CLIC3pCAF\#1} = 42$. $n_{CtlpCAF\#2} = 38$, $n_{CLIC3pCAF\#2} = 37$. n = HUVEC-coated beads assessed from three technical replicates of one biological replicate. P-values according to Mann-Whitney test. (f) Proliferation of HUVEC treated for 48h with conditioned medium (CM, EGM-2) generated from iCAF silenced for CLIC3 with two independent siRNAs or with non-targeting siRNA, measured by means of % of cells which incorporated EdU (= cells in S-phase). N = 8 (siCtl_2) or 9 measurements from three technical replicates. (g) Western blot analysis for CLIC3 showing that iNF stably carrying retroviral vector with CLIC3 engineered to contain a signal peptide, but not when carrying the empty retroviral vector (EV), express CLIC3 and that this is found in the total cell lysate (top) and in the conditioned medium (CM, bottom). (h) Sprout number quantification of HUVEC in 3D fibrin gel co-cultured with iNF and treated with the indicated amount of rCLIC3. $n_0 = 548$, $n_{0.25} = 246$, $n_{2.5} = 214$, $n_{25} = 250$, $n_{250} = 258$. n = HUVEC-coated beads assessed from two biological replicates. (i) Representative bright field images and quantification of mouse aortic rings treated with VEGFA (30 ng/ml) and vehicle (Ctl) or rCLIC3 (25 ng/ml). N = rings obtained from 4 aortas. Bars = mean +/- SEM.

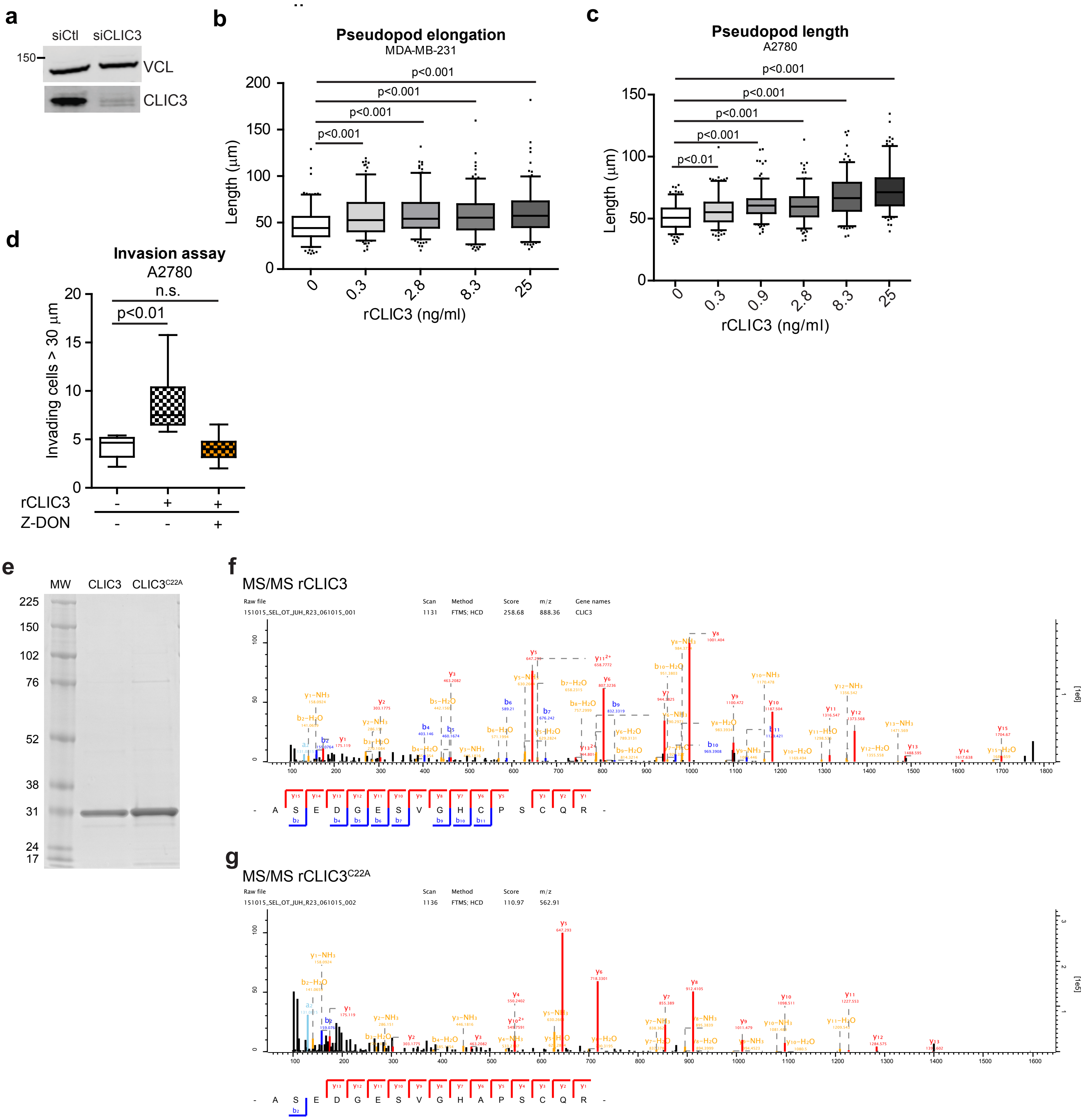

Supplementary Figure 5. CLIC3 cooperates with TGM2. (a) Western blot analysis for CLIC3 showing the efficient silencing upon transfection of iCAF with siRNAs specific for CLIC3. These cells were used for the invasive pseudopod length assay reported in Fig. 4e. (b,c) Quantification of the invasive pseudopod length of MDA-MB-231 breast (b) and A2780 ovarian (c) cancer cells migrating on cell-free ECM produced by telomerase immortalized fibroblasts (TIF) and treated with the indicated amount of rCLIC3. Whiskers plot (5-95 percentile) = mean +/- SEM. $n_{MDA\text{-}MB\text{-}231}$ = 180 cells assessed in one experiment, n_{A2780} = 540 cells assessed in three biological replicates. P-value according to Kruskal-Wallis test corrected with Dunn's test. (d) Quantification of A2780 cells invading into Matrigel plug embedded with GST (Ctl) or rCLIC3 (25 ng/ml), in the presence or absence of Z-DON (20 nM). Whiskers plot (5-95 percentile). n = 36 (except for the Ctl where n = 34). n = fields assessed from four biological replicates. (e) Gel stained with Comassie blue showing the purified recombinant CLIC3 wild type (rCLIC3) or where the cysteine 22 has been replaced by an alanine (rCLIC3^{C22A}). (f,g) Annotated MS/MS spectra (as visualised with the Viewer module of MaxQuant) obtained by MS analysis of the bands cut and digested from the gel shown in (e) which show that rCLIC3 has a cysteine in position 22 (f) while rCLIC3^{C22A} has an alanine (g).

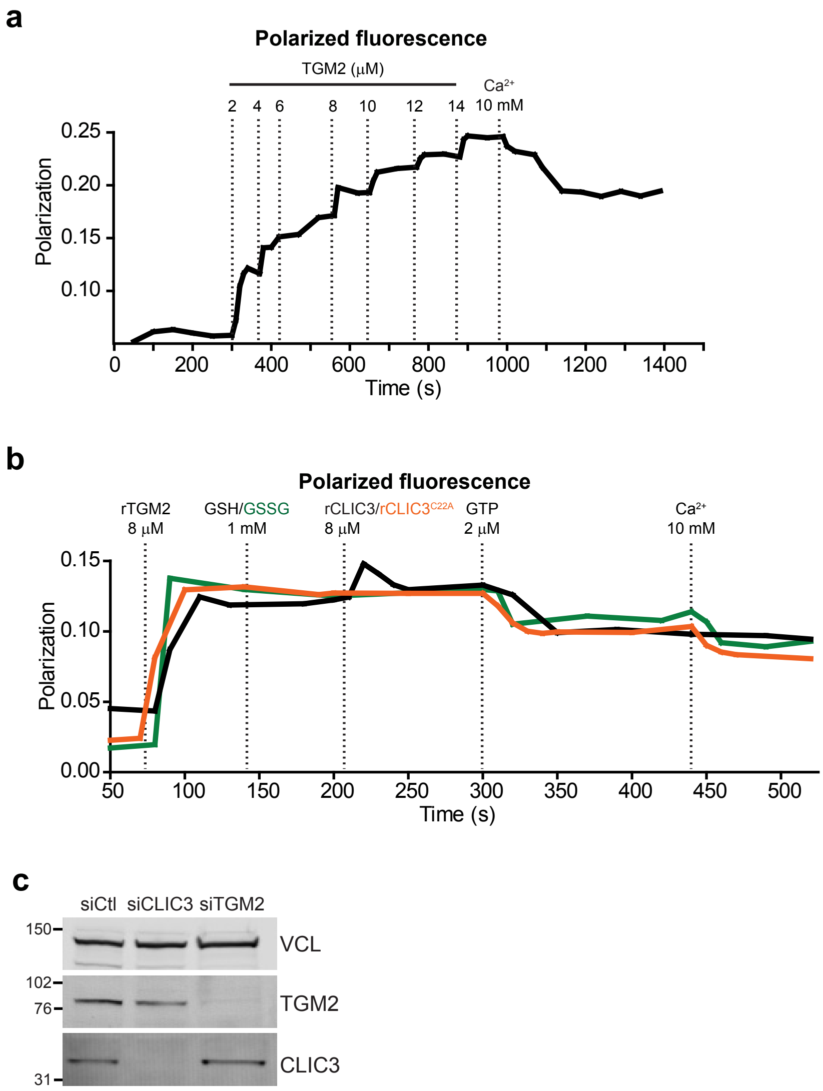

Supplementary Figure 6. CLIC3 controls TGM2 binding to its cofactors. (a) Polarized fluorescence measurements using Mant-GMPPNP and titrating in increasing amounts of rTGM2 followed by the addition of Ca^{2+}. (b) Polarized fluorescence measurements using Mant-GMPPNP and step wise addition of rTGM2, GSH or GSSG, and rCLIC3 or rCLIC3^{C22A} followed by the addition of unlabelled GTP and Ca2+ as indicated on the figure. Same plot as in Fig. 5d, but with the y axis starting from zero. (c) Western blot analysis for CLIC3 and TGM2 showing the efficient silencing upon transfection of iCAF with siRNAs specific for CLIC3 and TGM2. These cells were used for the production of the cells used in Fig. 5e.

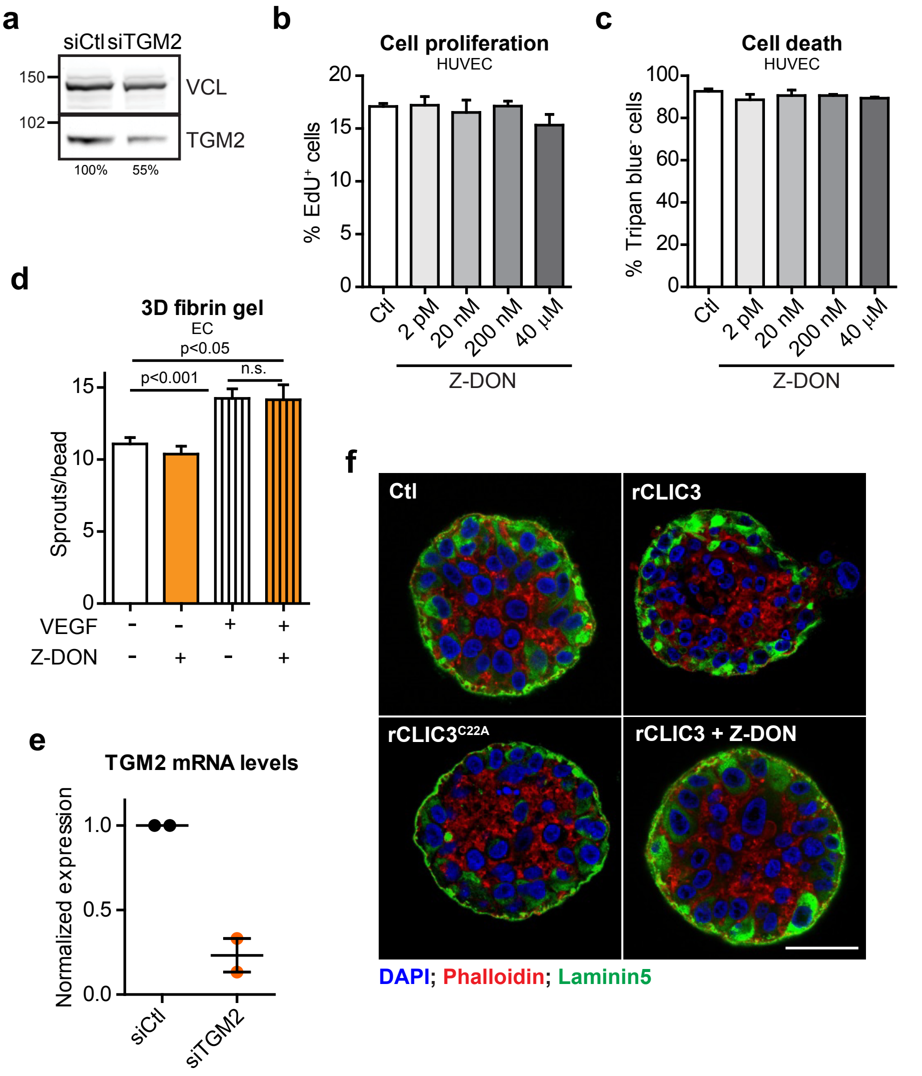

Supplementary Figure 7. Extracellular CLIC3 requires TGM2 to drive invasion. (a) Western blot analysis for TGM2 showing the efficiency of the silencing with siRNA specific for TGM2 in HUVEC used for the 3D fibrin gel assay in Fig. 6a. TGM2 quantification (shown as % of TGM2 compared to siCtl) based on LICOR software. (b) Proliferation of HUVEC treated with Z-DON for 48h at the indicated concentration, measured by means of % of cells which incorporated EdU (= cells in S-phase) over an incubation time of 2h. Bars represent mean ± SEM (n=3 technical replicates). Results are representative of two biological replicates. (c) HUVEC cell death measured by means of % of cells which did not incorporate trypan blue (Life Technologies). Incorporation was measured by Countess Automated Cell Counter (Life Technologies) on cells harvested after 48h treatment with Z-DON at the indicated concentration. Bars represent mean ± SEM (n=3 technical replicates). Results are representative of two biological replicates. (d) Sprouting quantification of HUVECs in 3D fibrin gel stimulated for 2 days with vehicle (-), VEGFA (50 ng/ml) and/or Z-DON (20 nM). n_{Ctl} = 34, $n_{Z\text{-}DON}$ = 45, n_{VEGF} = 33, $n_{VEGF+Z\text{-}DON}$ = 27. n = HUVEC-coated beads assessed. Results are representative of two biological replicates. (e) Quantitative PCR analysis showing the efficiency of TGM2 silencing in telomerase-immortalized fibroblasts (TIF) which were used to generate the ECM for the pseudopod elongation experiment in Fig. 6c. TGM2 intensity has been first normalized to the intensity of the housekeeping gene GAPDH and then to siCtl (siCtl = 1). TGM2 quantification is shown as mean +/- SD (n = 2 independent experiments). (f) Representative immunofluorescence staining for Laminin-5 and actin (phalloidin) of comedo-like DCIS structure formed by MCF10DCIS.com cells cultured for 6 days in Matrigel, which formed in the presence of GST (Ctl, 25 ng/ml) or rCLIC3/rCLIC3^{C22A} (25 ng/ml), in the presence or absence of Z-DON (20 nM, ZD). Bar = 40 µm. Bars = mean +/- SEM.

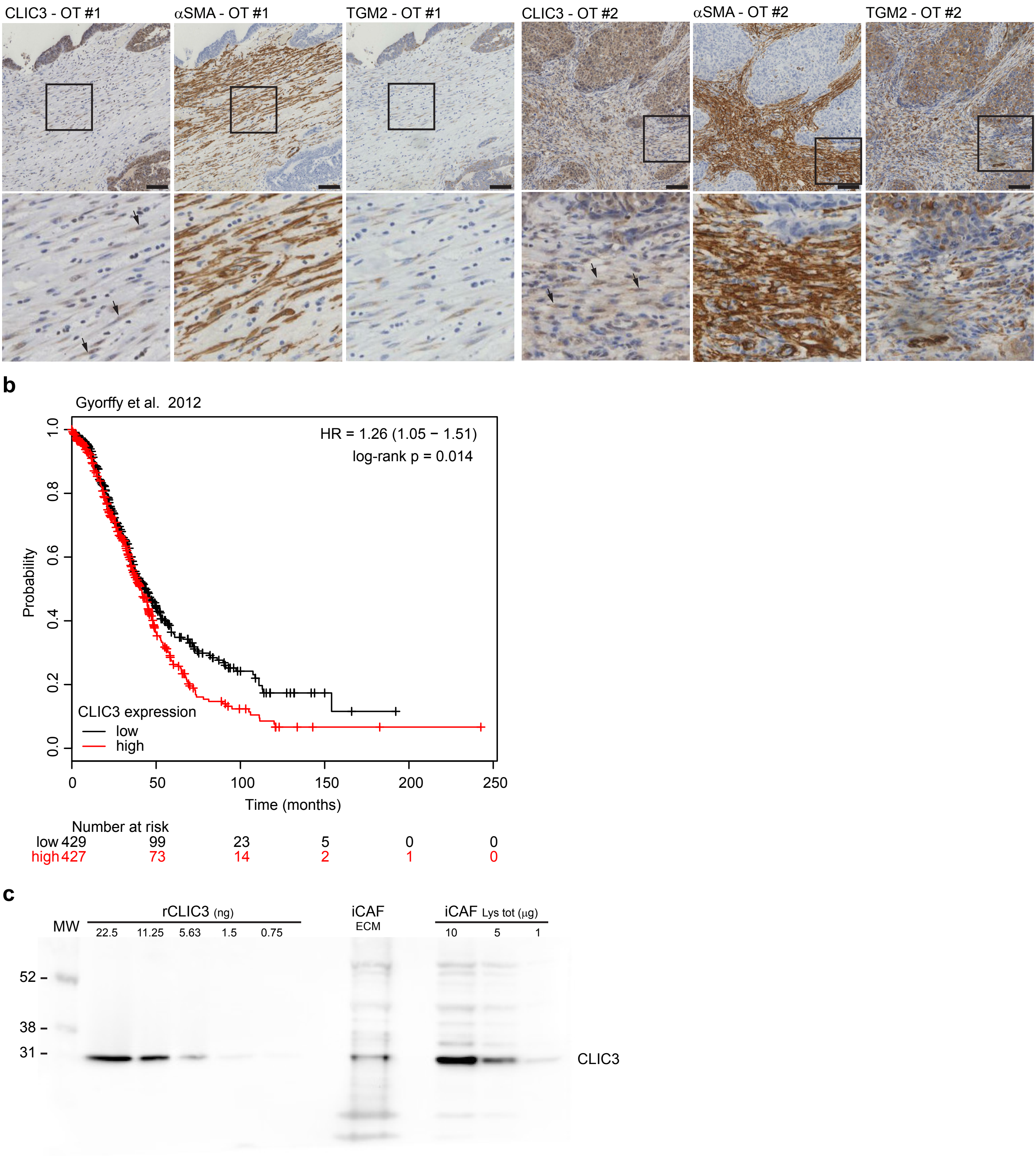

Supplementary Figure 8. High CLIC3 levels associates with ovarian cancer clinical outcome. (a) Immunohistochemistry for CLIC3, TGM2 and αSMA on ovarian tumour (OT) tissue sections obtained from two patients (#1 and #2). Arrowheads point CLIC3-positive cells in the stroma. Bottom panels represent the squared region highlighted in the top panels. Bar = 100 µm. (b) Kaplan–Meier analysis indicates that patients with high grade serous ovarian cancer with high CLIC3 gene expression (as reported in (5)) have poorer overall survival. (c) Western blot analysis for CLIC3 used to quantify the amount of CLIC3 in the total lysate and ECM deposited by iCAF. From a 6 cm dish of confluent iCAF cultured for 5 days, a total of 188 µg of proteins were extracted with SDS-containing lysis buffer. The amounts of total lysate loaded on the gel are indicated in the figure. For the ECM, proteins were extracted with SDS-containing lysis buffer from iCAF cultured confluent for 5 days in a 6 cm dish. One third of the extracted proteins were loaded on the gel. Bands were quantified with Image Studio Lite software, version 5.2.5; the total amount of CLIC3 estimated in the total cell lysate and ECM was around 480 ng and 25 ng, respectively.

Unedited full blots of Figure 1a

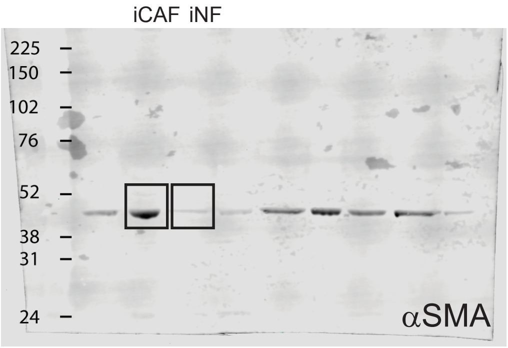

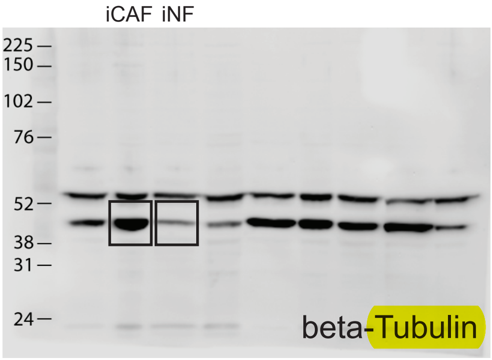

Unedited full blots of Figure 3a and 5a (total cell lysate)

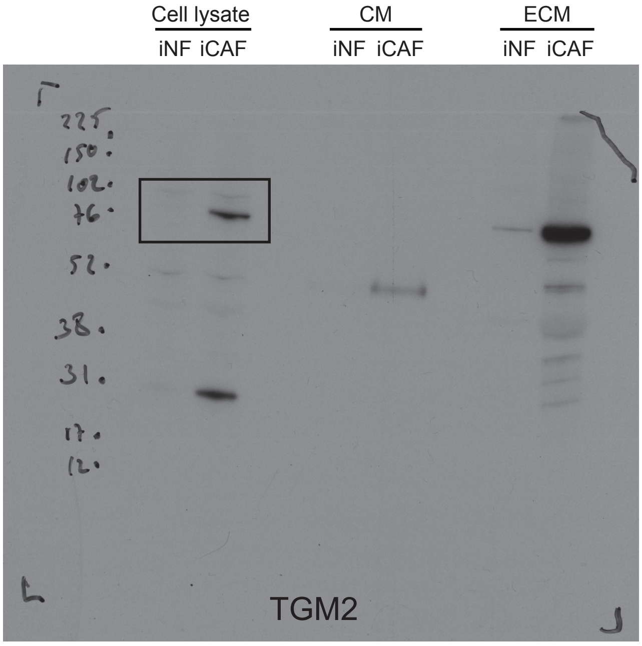

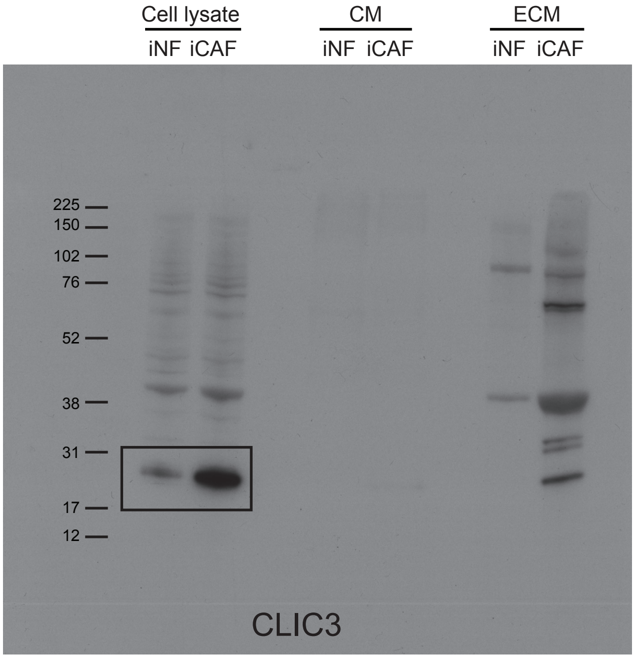

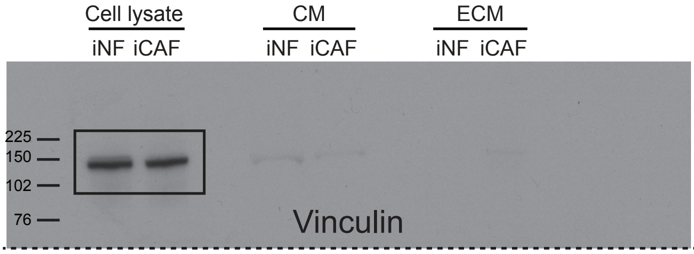

The original membrane was cut here before performing WB for Vinculin

Unedited full blots of Figure 3a and 5a (CM and ECM. Same samples as above but run on separated gel)

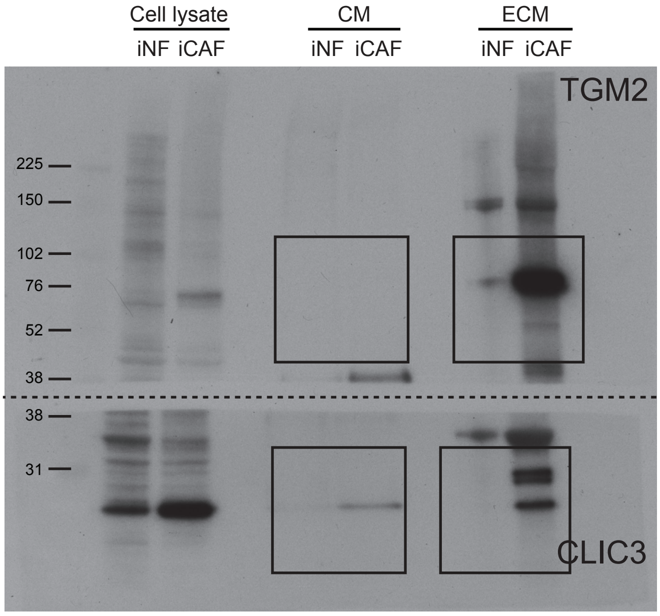

The original membrane was cut here before performing WB for CLIC3 and TGM2

Supplementary Figure 9. Unedited western blot

Unedited full blots of Figure 3b

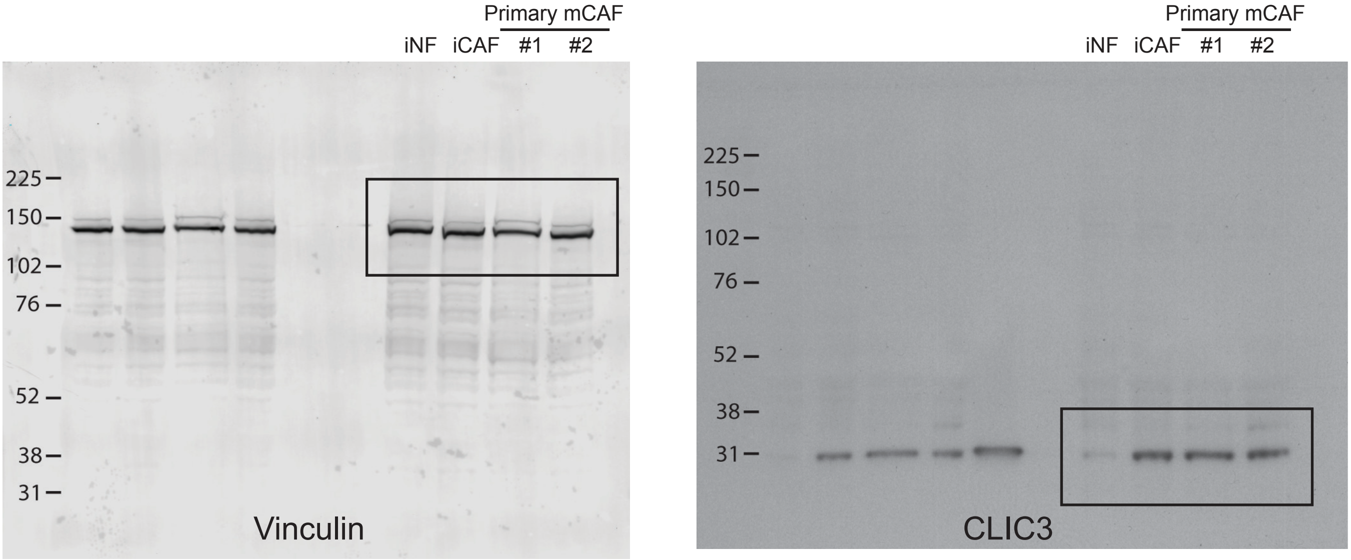

Unedited full blots of Figure 3c

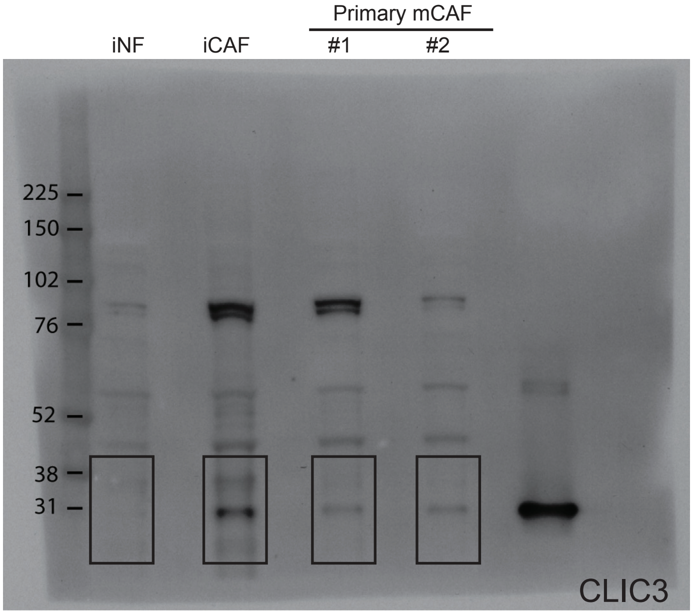

Unedited full blots of Figure 3d

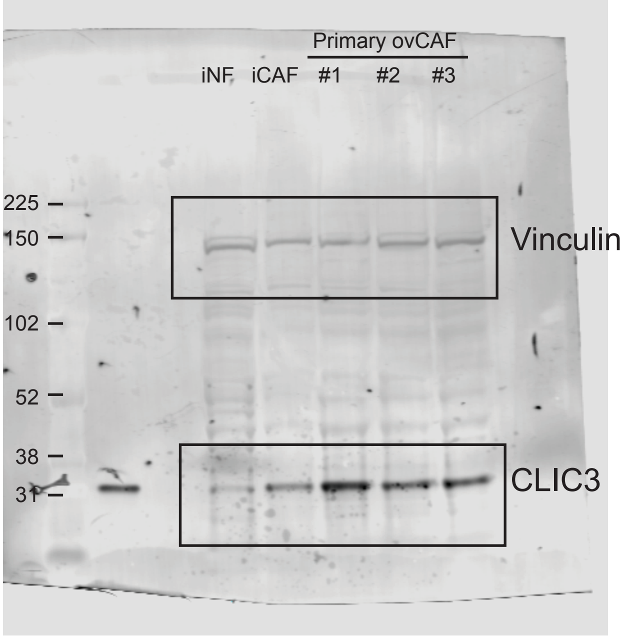

Unedited full blots of Figure 3e

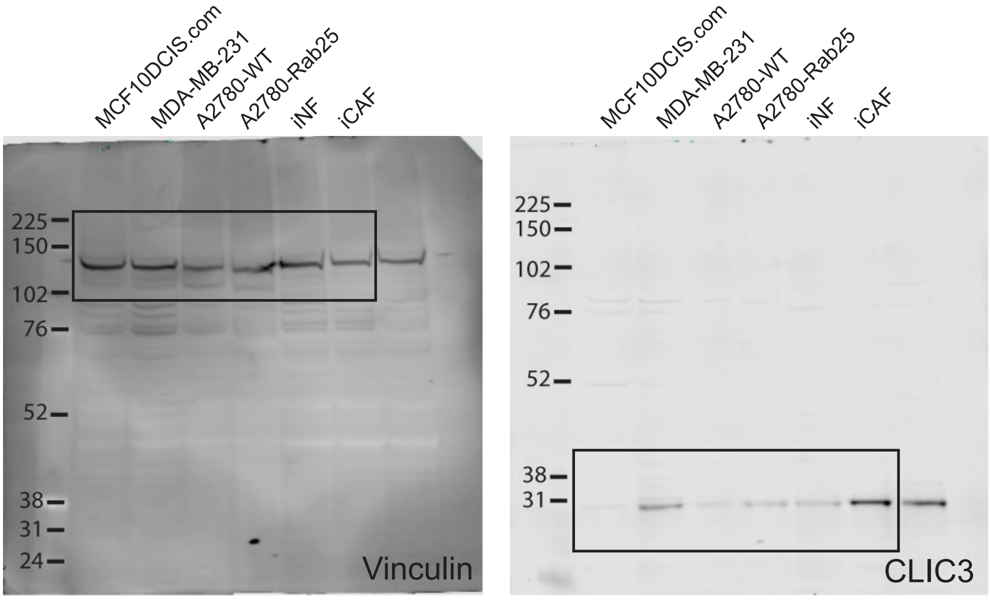

Unedited full blots of Figure 3f

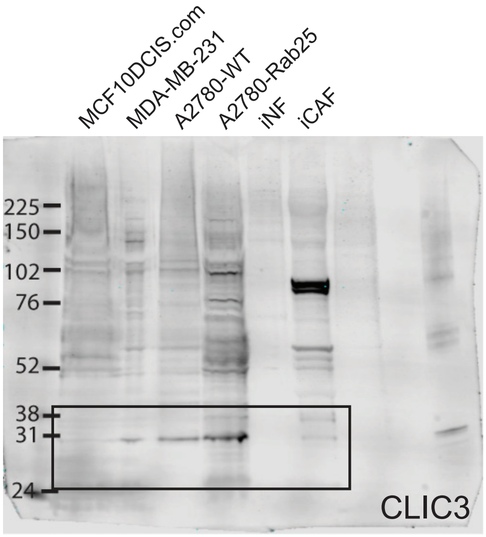

Supplementary Figure 10. Unedited western blot

Supplementary Table 1. Comparison iCAF signature with Bauer et al (Oncogene 2010)

Gene name	iCAF/iNF (log_2)	CAF/NF (log_2)
COL10A1	1.527	2.138
TGFB2	2.391	2.485
LYN	0.546	1.07
GALNT3	2.816	2.104
AKR1C1	-0.636	-1.415
PPAP2B	-1.752	-1.366
SLIT3	-1.595	-1.074

Supplementary Table 2. Comparison iCAF signature with Ma et al (Breast Cancer Res 2009)

Gene name	iCAF/iNF (log_2)	IDC/normal stroma (log_2)
COL8A1	3.564	4.9
FABP4	3.11	-4.4
SULF1	0.684	3.2
CTHRC1	0.263	3.5
FNDC1	0.179	5.1
COL12A1	-0.31	3.9
GREM1	-1.303	4.8
ASPN	1.56	4.3
COL10A1	1.527	6.25
RELN	-2.541	-3.5
SFRP1	1.462	-4
INHBA	0.91	4.4

Tumour type	CLIC3		αSMA	
	AVG	SD	AVG	SD
Ovarian Normal	-0.49	0.30	0.10	1.99
Ovarian Cancer	0.15	0.94	2.35	1.38
**Ovarian Cancer/Normal**	**0.64**		**2.25**	
Oral Normal	4.59	0.25	11.47	0.38
Oral Cancer	5.62	0.68	12.07	0.60
**Oral Cancer/Normal**	**1.03**		**0.60**	
Colorectal cancer Normal	-0.10	0.16	2.29	1.96
Colorectal cancer Tumour	0.64	0.99	3.23	1.24
**Colorectal Cancer/Normal**	**0.73**		**0.94**	

Supplementary Table 3. CLIC3 and αSMA mRNA levels (AVG = mean, SD = standard deviation) in normal and tumour stroma of ovarian (GSE40595), oral (GEOD-38517) and colon (GSE35602) carcinoma

Supplementary References

1. Bauer, M., *et al.* Heterogeneity of gene expression in stromal fibroblasts of human breast carcinomas and normal breast. *Oncogene* **29**, 1732-1740 (2010).
2. Casey, T., *et al.* Molecular signatures suggest a major role for stromal cells in development of invasive breast cancer. *Breast Cancer Res Treat* **114**, 47-62 (2009).
3. Ma, X.J., Dahiya, S., Richardson, E., Erlander, M. & Sgroi, D.C. Gene expression profiling of the tumor microenvironment during breast cancer progression. *Breast Cancer Res* **11**, R7 (2009).
4. Cline, M.S., *et al.* Integration of biological networks and gene expression data using Cytoscape. *Nature protocols* **2**, 2366-2382 (2007).
5. Gyorffy, B., Lanczky, A. & Szallasi, Z. Implementing an online tool for genome-wide validation of survival-associated biomarkers in ovarian-cancer using microarray data from 1287 patients. *Endocr Relat Cancer* **19**, 197-208 (2012).
